# Excessive postnatal smooth muscle differentiation in a lung-specific model of *TBX4*-related pulmonary hypertension

**DOI:** 10.1172/jci.insight.194251

**Published:** 2026-04-23

**Authors:** Lea C. Steffes, Kaylie A. Chiles, Sehar R. Masud, Aleen Rahman, Madeline Dawson, Csaba Galambos, Maya E. Kumar, Ripla Arora

**Affiliations:** 1 Department of Pediatrics, Division of Pulmonary Medicine, Stanford University School of Medicine, Palo Alto, California, USA.; 2 Department of Obstetrics, Gynecology, and Reproductive Biology, and; 3 Institute for Quantitative Health Science and Engineering, Michigan State University, East Lansing, Michigan, USA.; 4 Michigan State University College of Osteopathic Medicine, East Lansing, Michigan, USA.; 5 Departments of Pathology and Pediatrics, University of Colorado School of Medicine and Children’s Hospital Colorado, Aurora, Colorado, USA.

**Keywords:** Cardiology, Pulmonology, Hypertension

## Abstract

Heterozygous *TBX4* variants are the second most common genetic cause of pediatric pulmonary hypertension (PH), yet mechanisms underlying *TBX4*-related lung disease remain poorly understood. This study developed a lung-mesenchyme-specific *Tbx4* loss-of-function (*Tbx4cKO*) mouse model that bypasses embryonic lethality to investigate this condition. Adult *Tbx4cKO* mice demonstrated significantly impaired pulmonary flow acceleration consistent with PH. Three-dimensional analysis of embryonic lungs revealed reduced lobe volumes and decreased distance between pleural edges and muscularized vessels. In adult *Tbx4cKO* lungs, we identified extensive vascular remodeling characterized by medial thickening and the extension of muscularized arteries into normally non-muscularized subpleural parenchymal zones. Contrary to previous reports suggesting vascular simplification, 3-dimensional analysis demonstrated an elaborated pulmonary artery tree in addition to pathologic wall muscularization. Depletion of a single *Tbx5* allele in addition to both *Tbx4* alleles exacerbated histologic phenotypes, with worsened right ventricular dilation. This model also demonstrated dysregulated airway smooth muscle patterning and prominent subpleural smooth muscle bands, similar to those in human TBX4 syndrome. We identify TBX4 as a critical regulator of smooth muscle differentiation and patterning across multiple lung compartments. Our model recapitulates key features of human TBX4 syndrome and identifies dysregulated smooth muscle differentiation as a potential future therapeutic target.

## Abstract 2

Using a *Tbx4*-deficient mouse model we show that excessive and ectopic muscularization of the lung may underlie pulmonary hypertension in TBX4 syndrome patients

## Introduction

Pulmonary hypertension (PH) is a devastating and incurable disease defined by elevated pulmonary arterial pressures of greater than 20 mmHg (1). PH is now the leading indication for pediatric lung transplant (2, 3). Advanced PH is hallmarked by arterial changes, including increased thickness of the medial layer, muscularization of distal vessels, and the formation of occlusive luminal lesions (4). PH is driven by diverse upstream etiologies, including genetic mutations, cardiopulmonary comorbidities, systemic inflammatory conditions, drug and toxin exposures, and infections, and many cases remain idiopathic (1).

In pediatrics, over the past decade, the increased use of genetic testing has identified novel genetic causes of PH (5). Current research suggests that approximately 40% of children initially diagnosed with idiopathic pulmonary arterial hypertension (PAH) likely have an underlying disease-causing variant (6). These genetic discoveries provide a new lens through which to investigate the molecular mechanisms underlying vascular remodeling in this devastating disease. This deeper understanding could reveal shared and variant-specific pathways, enabling the future development of more effective therapeutics that can either target common disease mechanisms or be precisely tailored to specific genetic variants. Among these genetic causes, heterozygous T-box transcription factor 4 (*TBX4*) variants are particularly prevalent in pediatric-onset PAH, accounting for an estimated 8% of previously idiopathic cases, second only to bone morphogenetic protein receptor type 2 variants (6, 7). TBX4 syndrome case series encompassing adolescent and adult-onset PH indicate *TBX4*-related PH can manifest later in life (8–10). The true prevalence of *TBX4* variants in adult PH populations may be underestimated, as genetic testing is not routinely performed.

*Tbx4* encodes a transcription factor expressed in the developing hindlimb, lung, and umbilicus in mice (11, 12). Single-cell analysis has confirmed parallel expression patterns in the human developing hind limb and lung, although umbilical expression in humans remains to be determined (13, 14). *TBX4* variants were first recognized in ischiocoxopodopatellar syndrome (15), also known as small patella syndrome, with more recent recognition of lung and pulmonary vascular phenotypes (16, 17). A variable combination of PH, developmental lung disease, and hind limb abnormalities are now referred to as TBX4 syndrome (18). In patients with *TBX4*-related PH, the age of disease onset shows remarkable variability, ranging from fatal neonatal presentations to geriatric-onset disease (8, 9, 19, 20). TBX4 syndrome can manifest with a biphasic clinical picture in which transient neonatal PH and hypoxemia is followed by a “honeymoon” period of clinical wellness before the onset of chronic and progressive PH later in life (20). Since lung biopsies are rarely obtained in infants with resolving neonatal PH, there is limited insight into the extent of developmental vascular abnormalities present during this early phase and whether they persist subclinically during the apparent recovery phase. Classic PAH vascular changes are reported in chronic PH TBX4 syndrome patients of all ages, with case reports suggesting a worsening vascular phenotype over time (9, 20, 21). Alongside the vascular phenotype, histologic analysis of TBX4 syndrome lungs shows heterogeneous and patchy developmental lung disease, ranging from acinar dysplasia to mild alveolar growth abnormality, as well as ectopic smooth muscle cell (SMC) and even intrapulmonary metaplastic bone formation (19–21). It is incompletely known how loss of *TBX4* gene function causes this progressive, wide ranging, and distinct lung phenotype (18). A better molecular and cellular understanding of this process could inform clinical interventions to halt disease progression.

In both the human and mouse developing lung, *TBX4*/*Tbx4* is expressed exclusively in the mesenchyme (11, 22–25) and in mice it has been shown to mark the progenitors of vascular SMCs, airway SMCs, fibroblasts, pericytes, and chondrocytes (26, 27). No expression is detected in epithelial or endothelial lineages in either fetal human (13) or mouse single-cell datasets (25, 28). Postnatally, *TBX4* transcription remains mesenchymal, with expression in SMCs, pericytes, and fibroblasts (24, 25, 28, 29). This mesenchyme-specific localization of *TBX4* in the lung is distinct from most genes associated with PH, which are predominantly expressed in the endothelium (5). Loss of TBX4, a quintessential mesenchymal transcription factor, impacts developmental lineages that provide critical signaling and structural support to airways and vasculature both during embryogenesis and postnatally (12). This disruption aligns with the characteristic pathobiology of *TBX4*-associated lung disease, which manifests as a spectrum of airway, parenchymal, and pulmonary vascular abnormalities (16).

A mouse model of TBX4 syndrome would provide a valuable tool for investigating *TBX4*-driven lung disease in vivo. However, studying the effects of *Tbx4* loss of function on lung pathogenesis is challenging due to the lack of a reported phenotype in heterozygotes and embryonic lethality of homozygous *Tbx4*-null mice due to failure of chorioallantoic fusion and formation of a functional placenta (30). Conditional deletion of *Tbx4* using the global *Rosa26*CreER suggests that *Tbx4*-depleted lungs are smaller at mid-gestation, but embryonic lethality precluded any postnatal or adult analysis of lung phenotypes (22). To overcome these limitations, we developed a mouse model by combining a Cre recombinase whose expression is controlled by a lung-specific *Tbx4* enhancer (26) with *Tbx4* conditional floxed alleles (30), resulting in removal of *Tbx4* gene function throughout the lung mesenchyme and its derivatives. While this lung-specific deletion differs from human disease, where *Tbx4* is lost globally, it bypasses embryonic lethality and allows investigation of late embryonic, postnatal, and adult lung phenotypes. *Tbx5*, a homolog of *Tbx4*, displays overlapping expression with *Tbx4* and is critical for fetal lung development (22). Thus, a *Tbx5* conditional allele (31) was incorporated to examine the effects of loss of an additional T-box homolog on the lung phenotype.

Here, we show that mice with lung-mesenchyme-specific *Tbx4* deletion (*Tbx4cKO* mice) develop significantly impaired pulmonary flow acceleration consistent with PH coupled with airway and vascular changes matching those seen in TBX4 syndrome (Supplemental Table 1 and Supplemental Videos 1–3 [Control_parasternal short axis echo, Tbx4cKO_parasternal short axis echo, and Tbx4cKOTbx5het_parasternal short axis echo, respectively]; supplemental material available online with this article; https://doi.org/10.1172/jci.insight.194251DS1). Through quantitation of pulmonary artery (PA) remodeling from single optical sections, we identified significant medial thickening with *Tbx4* loss of function consistent with changes seen in human PH. Three-dimensional analysis of embryonic day 18.5 (E18.5) lungs revealed that *Tbx4*-mutant lungs have smaller lobe volumes and decreased distance between the pleural edge and muscularized arteries and airways. Similar 3-dimensional analysis of smooth muscle lineages in adult lungs revealed a progressive phenotype, with extensive distal muscularization of an elaborated artery tree, airway muscularization defects, widespread parenchymal myofibroblasts, and extensive subpleural SMC banding — all worsened with the additional loss of a *Tbx5* allele. Each phenotype features excessive smooth muscle deposition, suggesting a central function of lung mesenchymal *Tbx4* expression in appropriately regulating the formation of smooth muscle identities. This model of *TBX4*-related lung disease provides an invaluable tool for studying the mechanisms of PH development in TBX4 syndrome, enabling hypothesis generation and providing a tractable in vivo model for hypothesis testing.

## Results

### Mice lacking pulmonary Tbx4 gene function have PH.

To assess excision at the *Tbx4* locus, *Tbx4LMECre* semiquantitative PCR–based genotyping of entire lungs at E18.5 was used. DNA fragments were amplified for *Tbx4* wild-type, floxed (conditional), or null (excised) alleles (22). A reduction in the floxed band and a prominent null band indicates efficient excision at the *Tbx4* locus within the pulmonary mesenchyme (Supplemental Figure 1A). As endothelial and epithelial lineages do not express *Tbx4LMECre*, a band for the floxed allele is expected in both *Tbx4cKO* and control samples. *Tbx4cKO* pups were born in Mendelian ratios, with 50.8% of pups from the cross *Tbx4LMECre; Tbx4^fl/fl^* × *Tbx4^fl/fl^* inheriting Cre (*n* = 62/122 Cre-positive pups weaned from 12 litters). Equal proportions of males and females were born (29/62 Cre-positive pups were female and 33/62 pups were male) and adult *Tbx4cKO* mice had no differences in weight compared to control mice (Supplemental Figure 1B).

To further characterize the impact of fetal loss of *Tbx4* and *Tbx5* on adult lung function and the development of PH, we performed echocardiography on adult mice between 3 and 8 months of age (Supplemental Figure 2). PH metrics collected from mouse echocardiography include the ratio of pulmonary acceleration time to pulmonary ejection time (PAT/PET), a measurement of right ventricular (RV) afterload; a decrease in this value is correlated with increased severity of PH), RV and outflow tract (RVOT) dilation, and leftward deviation of the interventricular septum (measured by the RV-to–left ventricular area ratio, RV/LV) (32–34).

**Figure 1:**
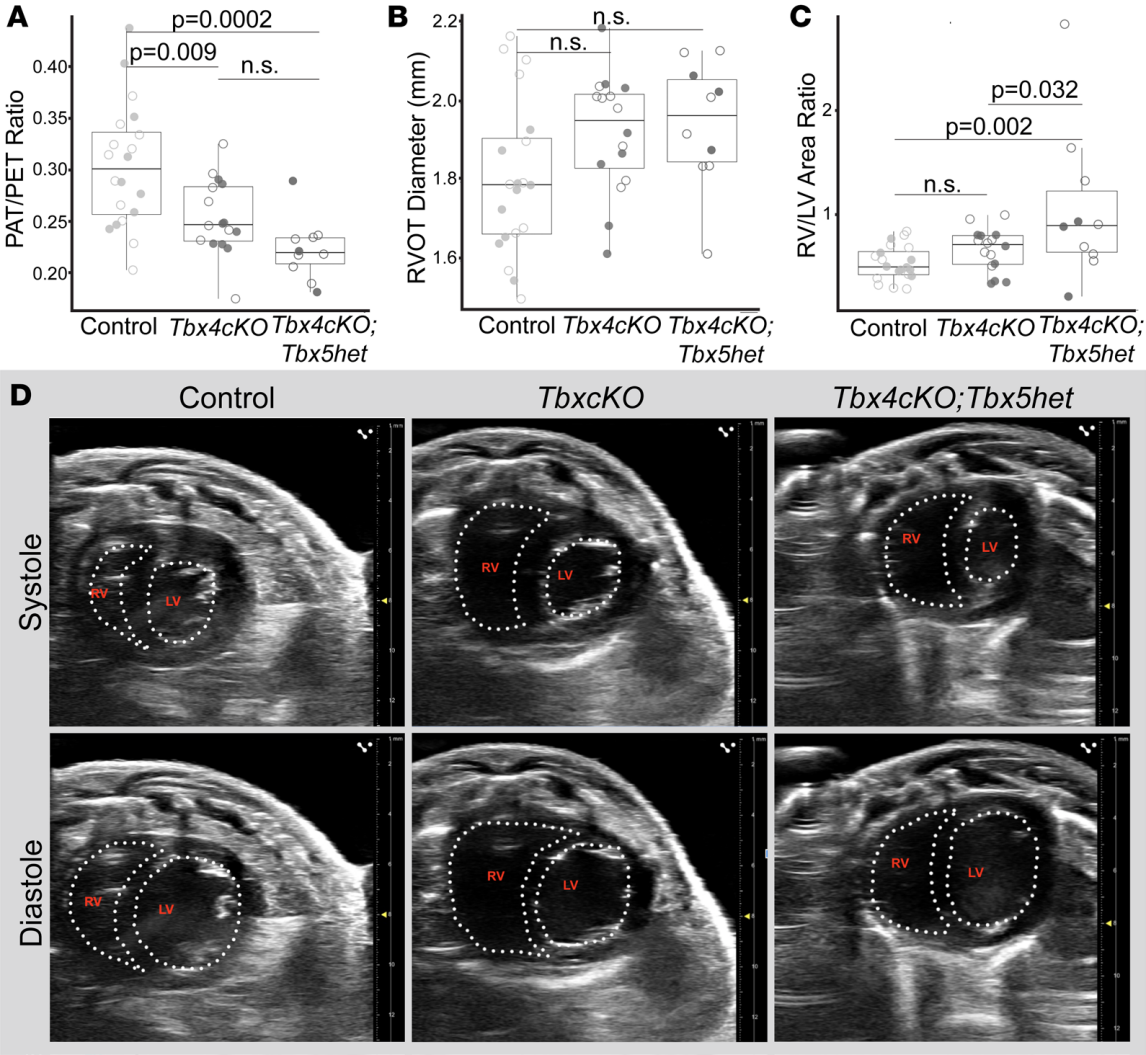
Echocardiographic assessment of adult mice infers PH in *Tbx4cKO* and *Tbx4cKO;Tbx5het*. (**A**) PAT/PET is significantly reduced in *Tbx4cKO* and *Tbx4cKO;Tbx5het* compared with control. There was no significant difference in RV outflow tract (RVOT) dilation (**B**) or RV/LV area ratio (**C**) in *Tbx4cKO*, although a significant increase was found in RV/LV area in *Tbx4cKO;Tbx5het* compared with control. *n* = 20 controls, *n* = 16 *Tbx4cKO*, *n* = 10 *Tbx4cKO;Tbx5het*. One-way ANOVA across genotypes, followed by Tukey’s HSD post hoc tests for pairwise comparisons between groups. Males, open circles; females, closed circles. (**D**) Representative images from systole and diastole phases of the cardiac cycle depicting the dilated RVs in *Tbx4cKO* and *Tbx4cKO;Tbx5het* mice, with flattening of the interventricular septum. Dotted lines denote the ventricular lumen.

*Tbx4cKO* adult mice exhibited a significant reduction in PAT/PET compared with controls (Figure 1A; *n* = 20 controls and *n* = 16 *Tbx4cKO*; *P* = 0.009 compared with control). Concurrent loss of one *Tbx5* allele (*Tbx4cKO;Tbx5het*) shows both reduced PAT/PET and statistically significant worsening of the RV/LV area (Figure 1, A–C; *n* = 10 *Tbx4cKO;Tbx5het;* PAT/PET *P* = 0.0002 compared with control and *P* = 0.243 compared with *Tbx4cKO;* RV/LV area *P* = 0.002 compared with control and *P* = 0.032 compared with *Tbx4cKO*). No statistically significant difference was seen in RVOT diameter between any groups (*P* = 0.065).

Representative short axis echo images by genotype are shown in Figure 1D. No significant changes in cardiac output (CO), stroke volume (SV), ejection fraction (EF), or fractional shortening (LV diameter change during systole) were observed between genotypes, indicating normal LV function (Supplemental Figure 2).

**Figure 2:**
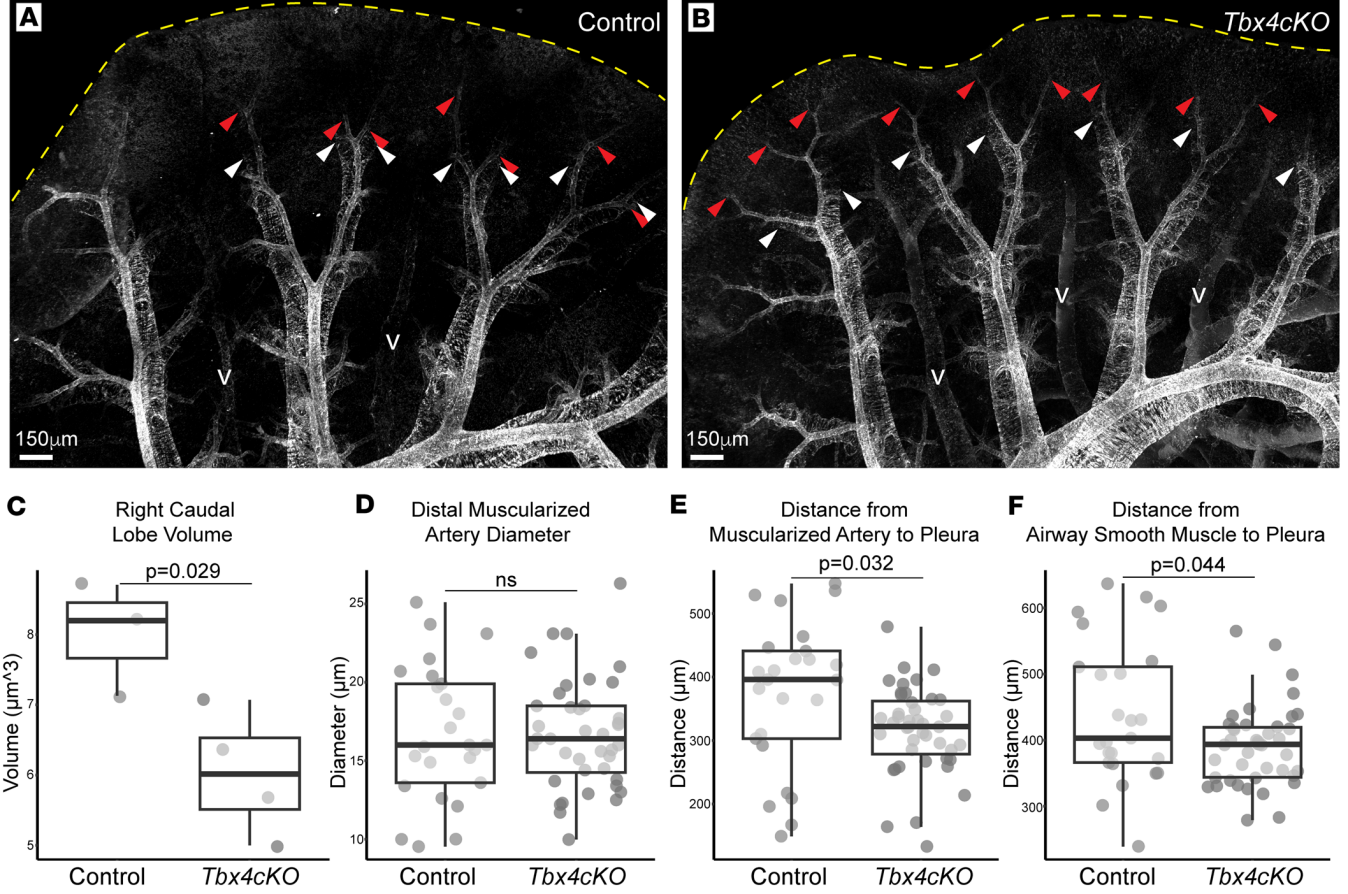
Decreased distance between muscularized airways and arteries and the pleura edge, as well as reduced lobe volume at E18.5 in *Tbx4cKO*. Confocal projections of whole right caudal lobes stained to highlight ACTA2 (white), cleared, and visualized in 3 dimensions from control (**A**) and *Tbx4cKO* (**B**), showing ACTA2^+^ airways and arterioles nearer to the pleural edge in *Tbx4cKO* compared with control at E18.5. White arrowheads mark distal most extent of airway smooth muscle; red arrowheads mark distal most coherent vascular smooth muscle; “V” marks veins. Note: The leftmost branch in **A** was excluded from quantitative analysis due to artifactual folding of the pleural edge during mounting. Scale bars: 150 μm. (**C**) Caudal lobe volume is significantly reduced in *Tbx4cKO* compared with control (*P* = 0.029). Distal muscularized artery diameter did not differ significantly between genotypes (**D**); however, the distance from distal-most vascular (**E**) and airway (**F**) smooth muscle to the pleural edge was significantly reduced in *Tbx4cKO* compared with control. *n* = 3 controls and *n* = 4 *Tbx4cKOs*. Welch’s ANOVA (assuming unequal variances) was performed to assess differences across genotypes, followed by Games-Howell post hoc tests for pairwise comparisons between groups.

*Tbx4cKO right caudal lobes at E18.5 are smaller with muscularized distal vessels and airway SMCs found close to pleural surface*. *TBX4* loss of function is associated with lethal developmental lung disorders hallmarked by hypoplastic lungs and varying degrees of acinar developmental failure (19). To identify changes at the end of embryogenesis, *Tbx4cKO* mice were harvested at E18.5, just before birth. Using whole-mount immunofluorescent staining, confocal microscopy, and image segmentation, we analyzed right caudal lobe volume and smooth muscle α-actin (ACTA2) expression around the distal arterioles and airways in uninflated lungs (Figure 2, A and B). Lobe volume was significantly smaller in *Tbx4cKO* mice compared with control (Figure 2C; average 6.02 × 10^9^ μm^3^ vs. 8.20 × 10^9^ μm^3^; *P* = 0.029) and a significant reduction in the distance between PA and airway SMCs and the pleura was observed in *Tbx4cKO* lobes compared with control. The shortest distance from the distal-most point of fully coherent arterial SMC coverage to the pleural edge was measured in arteries associated with the terminal bifurcations of the right caudal (RCd) lateral (L) branches 1-4 (RCd.L1 through RCd.L4) (35). PA diameter at the point of distal-most coherent PA SMC coverage was measured and no significant change was found in *Tbx4cKO* mice compared to control (Figure 2D; *P* = 0.97). *Tbx4cKO* mice exhibited a significant reduction in muscularized PA-to-pleura distances compared with control (Figure 2E; *P* = 0.032), with coherent PA SMC coverage terminating a median distance of 396 μm from the pleural edge in control lobes, and 322 μm in *Tbx4cKO*. In addition to distal PA SMC coverage, we assessed distal airway SMC coverage. Similar to PA SMCs, distal-most airway SMC coverage was significantly closer to the pleural edge in *Tbx4cKO* compared with control (Figure 2F; median of 403 μm in controls; 393.5 μm in *Tbx4cKO*; *P* = 0.044; *n* = 3 controls and *n* = 4 *Tbx4cKOs*).

### Tbx4cKO and Tbx4cKO;Tbx5het adult mice have significant medial thickening and rare neointimal formation.

**Figure 3:**
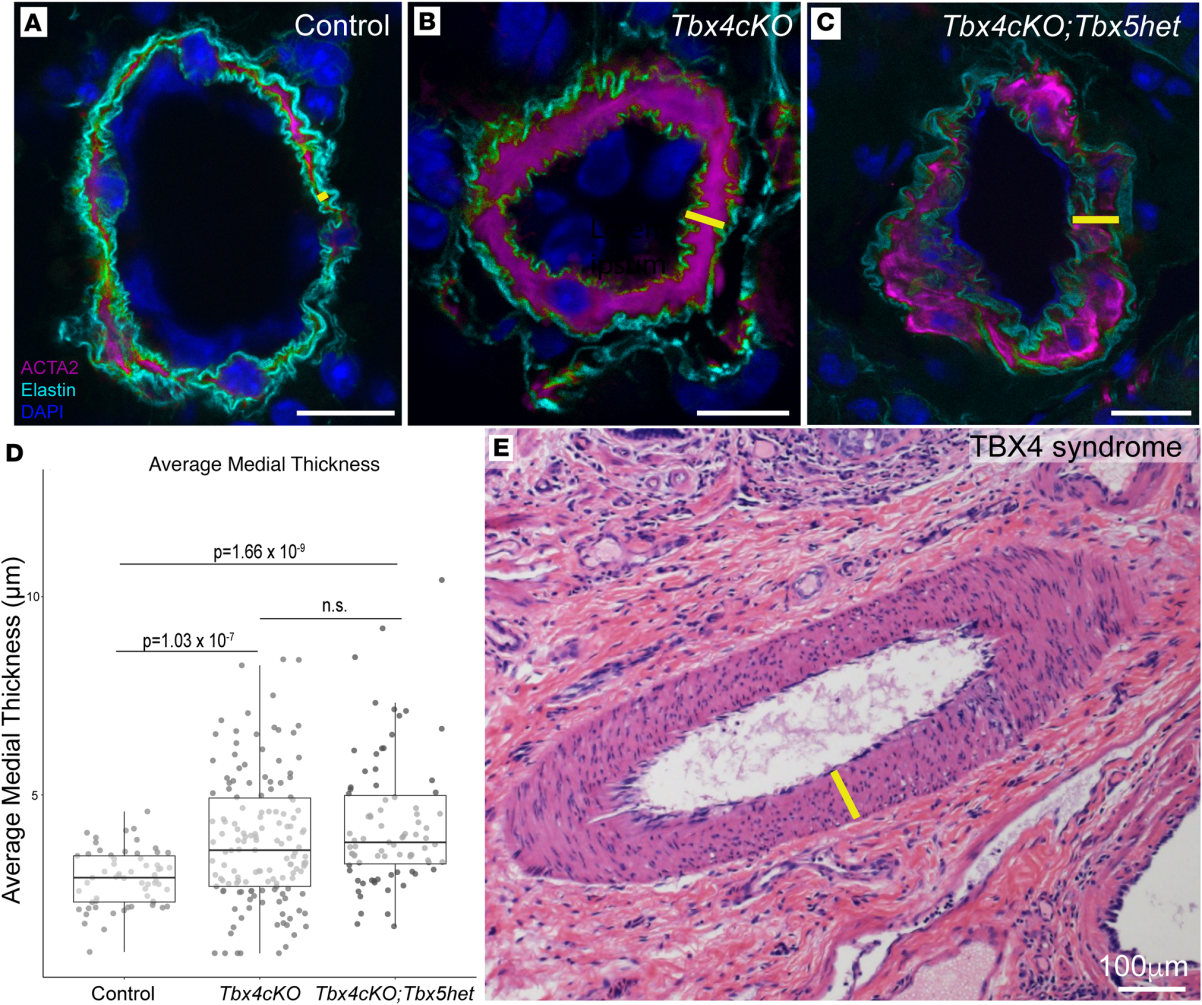
*Tbx4cKO* and *Tbx4cKO;Tbx5het* mice display pulmonary artery muscularization consistent with PH hemodynamics and similar to that seen in TBX4 syndrome. (**A**–**C**) Pulmonary artery cross sections from control, *Tbx4cKO*, and *Tbx4cKO;Tbx5het* mouse lungs stained to highlight media (ACTA2, magenta), internal and external elastic laminae (hydrazide, cyan), and nuclei (DAPI, blue) show an increase in medial thickness (yellow bars) with loss of *Tbx4* gene function from lung mesenchyme. (**D**) Measurement of medial thickness from high-resolution confocal images of individual arteries from each genotype confirms medial hypertrophy with loss of *Tbx4*. The additional loss of 1 copy of *Tbx5* does not lead to significant worsening of the phenotype. *n* = 3 for each genotype. Welch’s ANOVA (assuming unequal variances) was performed to assess differences across genotypes, followed by Games-Howell post hoc tests for pairwise comparisons between groups. (**E**) Pulmonary arteries (H&E stain of paraffin sections) of TBX4 syndrome patients display a similar expansion of the medial layer (yellow bar) versus control (see Supplemental Figure 3). Scale bars: 10 μm (**A**–**C**) and 100 μm (**E**).

Increased muscularization and arterial remodeling is seen with *TBX4*-related pulmonary vascular disease (9, 20, 21) (Figure 3E and Supplemental Figure 3). Thickening of the smooth muscle media, located between the internal and external elastic lamina, and formation of neointimal lesions, which are cellular accumulations between the intima and internal elastic lamina, are hallmarks of human PAH histology and drive PH hemodynamics (4). To uncover the cause of elevated PA pressures observed in adult *Tbx4cKO*, we performed a detailed examination of PA anatomy in these animals (Figure 3, A–C). Although average PA diameter was comparable between genotypes (56.7 μm in control vs. 55.9 μm in *Tbx4cKO*), media layers were significantly thicker in *Tbx4cKO* animals, with an increase in mean thickness of 24% compared with controls (Figure 3D; 3.6 μm in *Tbx4cKO* and 2.9 μm in control, *P* = 1.03 × 10^–7^). There was no significant increase in medial thickness in *Tbx4cKO;Tbx5het* compared to *Tbx4cKO* (*P* = 0.11). Consistent with medial thickening reducing the size of the patent vessel lumen, percentage lumen (vessel diameter minus medial thicknesses divided by vessel diameter) was significantly reduced in *Tbx4cKO* compared with control (Supplemental Figure 4A; *P* = 2.88 × 10^–5^) and *Tbx4cKO;Tbx5het* (*P* = 0.01 vs. control). There was no significant difference between *Tbx4cKO* and *Tbx4cKO;Tbx5het*. In one *Tbx4cKO* animal, frequent, partially occlusive neointimal lesions were observed (neointima present in 38% of measured arteries; 11 of 29 arteries scored), but neointima was largely absent in all other mutant animals evaluated (neointima present in 0.31% of all remaining arteries; 1 of 318 arteries scored; *n* = 3 for each genotype). Similar rare neointimal changes are observed in human TBX4 syndrome lung histology (20).

### Whole-mount analysis of Tbx4cKO and Tbx4cKO;Tbx5het lungs demonstrates excessive vascular SMC coverage and distal muscularization of small arteries.

**Figure 4:**
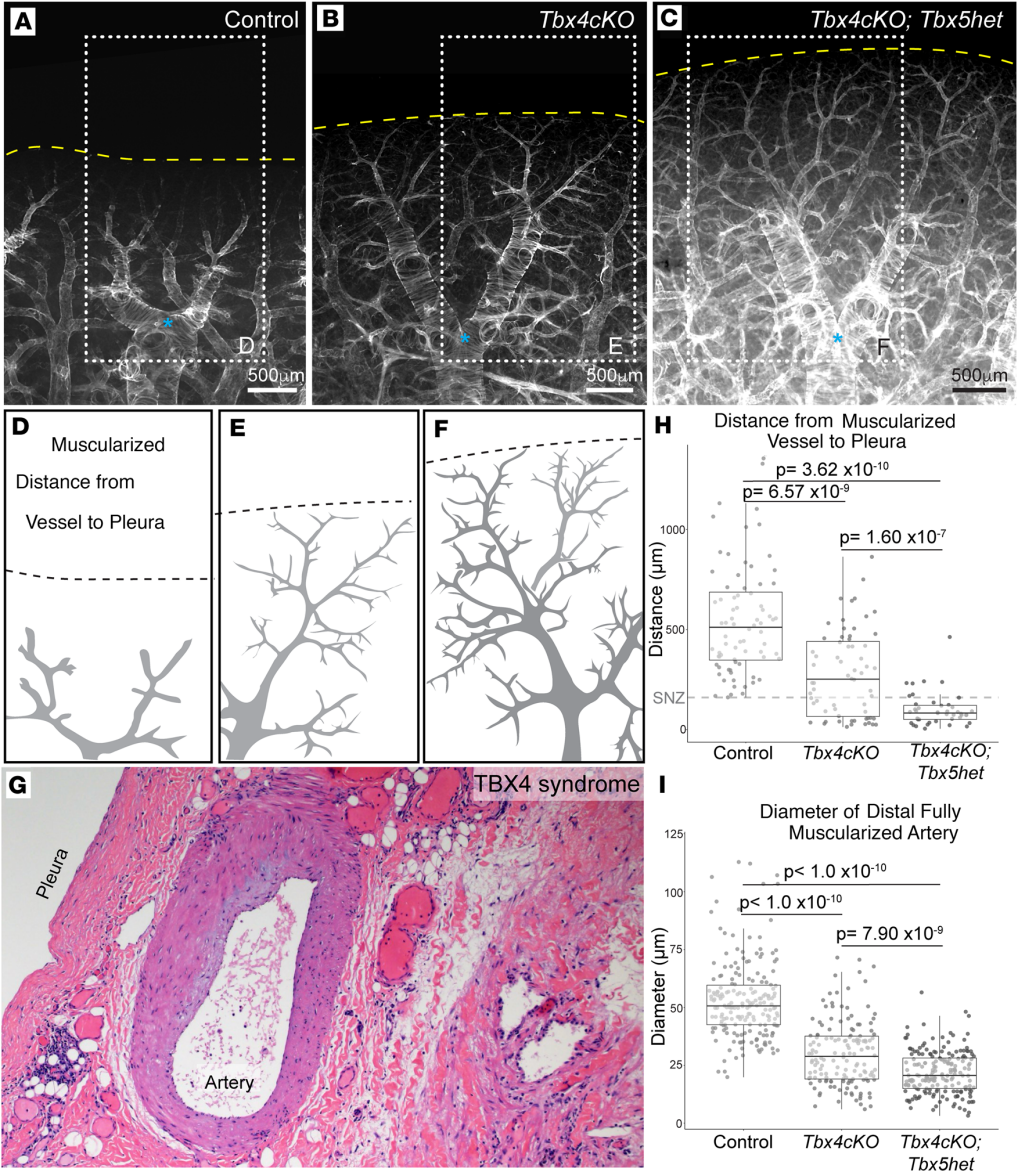
Whole-mount analysis reveals increased vascular smooth muscle coverage of peripheral pulmonary artery networks in *Tbx4cKO* and *Tbx4cKO;Tbx5het*. (**A**–**C**) Intact right caudal lobes stained to highlight smooth muscle α-actin (ACTA2) protein (white), cleared, and visualized in 3 dimensions from control, *Tbx4cKO*, and *Tbx4cKO;Tbx5het* animals allows visualization of both airway, arterial, and venous smooth muscle networks. The same branch (dorsal view of RCd.L3) is shown for each genotype with distal-most airway bifurcation marked with asterisk to aid comparison. Images in **A**–**C** are multifocal projections of stereoscope *z*-stacks. Scale bars: 500 μm. (**D**–**F**) Schematic drawings of arterial networks for each genotype. The gray dotted line in **E** at 160 μm demarcates the subpleural nonmuscular zone (SNZ) where muscularized pulmonary arteries are absent in control lungs. (**G**) Highly muscularized arteries (H&E stain of paraffin section) are found close to the pleural surface in TBX4 syndrome lungs but not in control (see Supplemental Figure 3). (**H**) In both *Tbx4cKO* and *Tbx4cKO;Tbx5het*, muscularized arteries extend significantly closer to the pleura (yellow dotted lines in **A**–**C**; black dotted lines in **D**–**F**). (**I**) Consistent with abnormal smooth muscle coverage of slender distal pulmonary arteries, the diameter of the distal-most fully muscularized arteries drops significantly in both *Tbx4cKO* and *Tbx4cKO;Tbx5het* animals. Control *n* = 7, *Tbx4cKO* *n* = 5, *Tbx4cKO;Tbx5het* *n* = 3. Welch’s ANOVA (assuming unequal variances) was performed to assess differences across genotypes, followed by Games-Howell post hoc tests for pairwise comparisons between groups.

To assess the distribution and extent of muscularized arteries with fine spatial precision, whole-mount ACTA2 antibody staining, deep tissue imaging, and detailed quantitation of intact right caudal lobes were performed. A striking feature of the mutant phenotype was the abnormal proximity of fully muscularized arteries to the pleural surface (Figure 4, A–F), paralleling observations in TBX4 syndrome in which muscularized arteries abut the pleura (Figure 4G). To confirm that this reflects true extension of smooth muscle coverage, both the distance from the pleural edge to the distal-most coherent SMC coverage and the length of SMC-covered arterial segments from defined proximal artery bifurcation points were measured (Figure 4H and Supplemental Figure 4B). As in the embryonic analysis above, to allow quantitative comparison between genotypes, we assessed the arteries accompanying the terminal bifurcations of airway branches RCd.L1 through RCd.L4 (35) in all animals. In control lungs, arteries have a coherent ACTA2^+^ media until a median distance of 511 μm from the pleura, after which only sparse, discontinuous ACTA2^+^ cells are observed. However, in *Tbx4cKO* mice, we found coherent muscularization extended a median distance of 278 μm (*P* = 6.57 × 10^–9^ vs. control) from the pleural edge (Figure 4H). Many fully muscularized arteries extended into what is normally a non-muscularized vascular zone, with 39.1% of PAs showing complete smooth muscle medial coat within 160 μm of the pleura in *Tbx4cKO* compared with zero in the control. We termed this region that is typically free of muscularized PA the subpleural non-muscularized zone (SNZ), demarcated as a dashed line in Figure 4H. In addition, *Tbx4cKO* arteries showed longer SMC-covered segments compared with controls (Supplemental Figure 4B, *P* = 0.014), again indicating that smooth muscle extends more distally along the arterial tree.

Distal muscularization, in which smaller diameter arteries have a complete SMC coat, is seen in PH histology and is thought to be an important driver of increased pulmonary vascular resistance (4). We assessed distal muscularization by measuring the diameter of arteries at the point where the complete SMC coat ends and found that smaller diameter arteries were fully muscularized in *Tbx4cKO* compared with controls (Figure 4I; median of 30.2 μm in *Tbx4cKO* vs. 53.1 μm in control; *P* < 1.0 × 10^–10^).

All PA muscularization parameters were exacerbated in *Tbx4cKO;Tbx5het*, with the distance to the pleura of muscularized arteries shrinking to a median of 100 μm (*P* = 1.60 × 10^–7^ vs. *Tbx4cKO*, *P* = 3.62 × 10^–10^ vs. control), the percentage of muscularized vessels within the SNZ increasing to 83.7% (Figure 4H), and the diameter of muscularized tips narrowing further to 21.9 μm (Figure 4F; *P* = 7.9 × 10^–9^ vs. *Tbx4cKO*, *P* < 1.0 × 10^–10^ vs. control; control *n* = 7, *Tbx4cKO n* = 5, *Tbx4cKO;Tbx5het* *n* = 3). Together, these findings indicate a key role for T-box genes in regulating proper distal arterial muscularization.

### Excessive PA branching and abnormal patterning of muscularized arteries in Tbx4cKO mice.

Arteries of all sizes have elastic laminae that disappear only as they transition to the capillary bed (36). Staining these elastin layers allows us to visualize the arterial tree in whole-mount samples independently of the presence of SMCs (Supplemental Figure 5). To allow direct comparison between control and mutant samples, we quantitated branch number and the presence of an SMC coat of the same PA (the PA serving the second ventral airway branch emerging from the first lateral branch of the right caudal lobe; RCd.L1.V2 [V, ventral]; branch number was determined by counting all PA branch points visible with either ACTA2 or elastin staining in the PA tree stemming from the first lateral branch) in control and *Tbx4cKO* mice. In control, distal arteries are ACTA2-negative, with 44% of elastin-marked PA branches lacking an SMC coat (26 elastin-marked branches, 15 ACTA2-coated). *Tbx4cKO* shows a more elaborate and muscularized arterial tree with a greater number of elastin-marked branches, all of which are invested with an SMC coat. In *Tbx4cKO*, the SMC coat extends beyond the visualizable elastic lamina, with 47 elastin-marked branches scored but 56 ACTA2-covered branches visualized. While the increased number of arterial branches might initially suggest increased vascular surface area that could reduce pulmonary vascular resistance, these are abnormal vessels with extensive distal muscularization of smaller diameter vessels, medial thickening, and luminal narrowing consistent with the observed echocardiographic evidence of PH.

### Airway muscularization extends closer to pleura in Tbx4cKO and Tbx4cKO;Tbx5het compared with control and myofibroblasts are abundant in Tbx4cKO.

**Figure 5:**
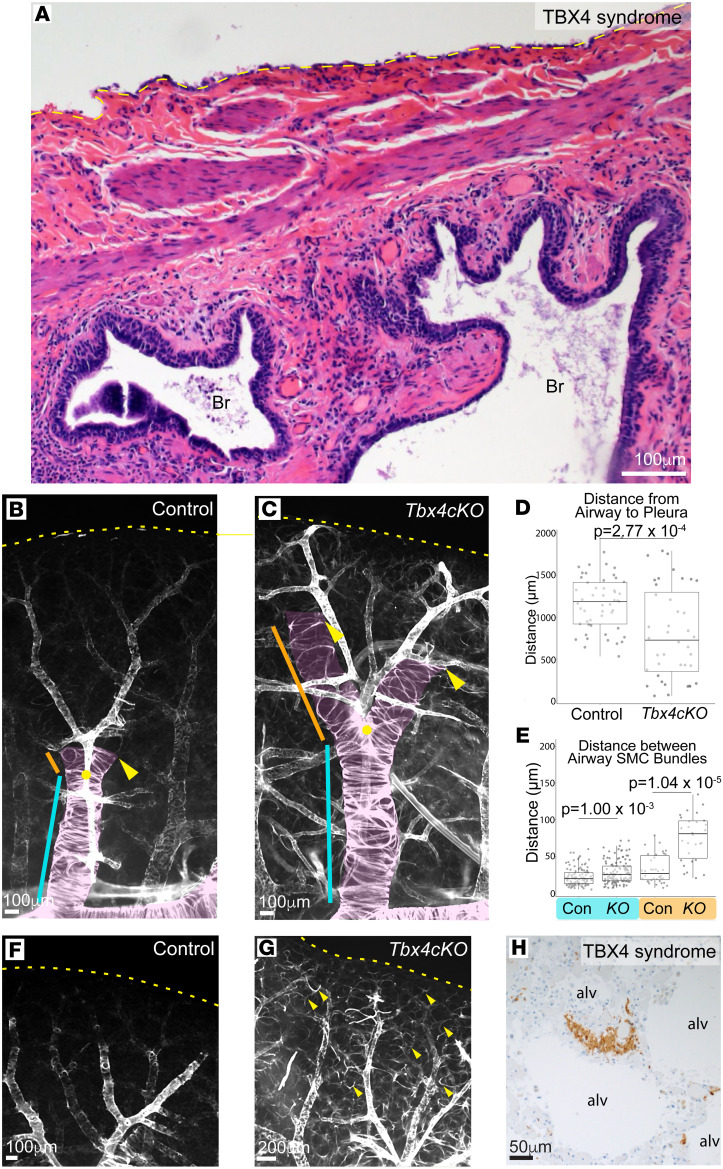
Airway smooth muscle extends closer to the pleura and is mispatterned in *Tbx4-*mutant lungs. (**A**) In TBX4 syndrome, smooth muscle–wrapped conducting airways are found in close apposition to the pleura, while control conducting airways end more proximally (see Supplemental Figure 3). (**B** and **C**) Whole-mount stains of right caudal lobes highlight ACTA2^+^ cells (white) along airways and vasculature. The same branch (dorsal view of RCd.L1.Cr2) is shown in each genotype, with airway-associated smooth muscle indicated with pink overlay and the position of the distal-most complete smooth muscle ring indicated with yellow arrowheads. (**D**) The distance between distal-most airway SMC ring and the pleural margin is significantly shorter in *Tbx4cKO* animals versus control. Control *n* = 7, *Tbx4cKO* *n* = 4. (**E**) The distance between airway smooth muscle bundles both before (cyan bar in **B** and **C**) and after (orange bar in **B** and **C**) the final conducting airway bifurcation (yellow dot in **B** and **C**) is significantly wider in *Tbx4cKO*. Con, control; KO, *Tbx4cKO*. Cyan, proximal measurements; orange, distal measurements. Control *n* = 3, *Tbx4cKO* *n* = 3. (**D** and **E**) Welch’s ANOVA (assuming unequal variances) was performed to assess differences across genotypes, followed by Games-Howell post hoc tests for pairwise comparisons between groups. (**F** and **G**) Whole-mount ACTA2 (white) staining of right caudal lobe margins shows an abundance of ACTA2^+^ C-shaped myofibroblasts (selected cells highlighted with arrowheads) in parenchyma of *Tbx4cKO* not present in control. Pleural lobe margin indicated with yellow dotted line in **A**–**C**, **F**, and **H**. ACTA2 immunostaining highlights a focus of ectopic smooth muscle accumulation in the lung interstitium in a patient with TBX4 syndrome (alv marks alveoli). Scale bars: 100 μm (**A**–**C** and **F**), 200 μm (**G**), and 50 μm (**H**).

Because bronchioles abutting the pleural edge are observed in TBX4 syndrome patients (Figure 5A) (20) we investigated the distribution of SMC-invested conducting airways in relation to the pleura in *Tbx4cKO* mice. In healthy animals, parallel rings of SMCs expressing ACTA2 wrap around conducting airways. These rings are formed by closely spaced bundles of SMCs (Figure 5, B and C). Moving from proximal to distal, as conducting airways transition to respiratory epithelium, the SMC rings abruptly become sparse and are absent in the most peripheral airways (37). In *Tbx4cKO* animals, airway SMC rings are present closer to the pleura than in control (Figure 5D; median of 731 μm in KO vs. 1188 μm in control, *P* = 2.77 × 10^–4^). Additionally, the spacing of SMC rings was altered in *Tbx4cKO*, with wider spacing of rings both proximally (median gaps of 25.6 μm in *Tbx4cKO* vs. 19.7 μm in control, *P* = 1.00 × 10^–3^) and distally (median gaps of 80.9 μm in *Tbx4cKO* vs. 25.6 μm in control, *P* = 1.04 × 10^–5^) relative to a fixed position in the airway tree, the terminal bifurcation of branches RCd.L1.Cr1, RCd.L1.Cr2, and RCd.L1.Cr3 (Cr, cranial) (35) (Figure 5E). Measurement of gap distances and the distal extent of airway SMC rings in *Tbx4cKO;Tbx5het* was unreliable due to the large number of ACTA2^+^ myofibroblast-like cells throughout the parenchyma (airway to pleura measurements: control *n* = 7, *Tbx4cKO* *n* = 4; SMC gap distance measurements: control *n* = 3, *Tbx4cKO* *n* = 3). In addition, dispersed C-shaped ACTA2^+^ cells not present in controls were found throughout the parenchyma of *Tbx4cKO* lungs (Figure 5, F and G) with further expansion in *Tbx4cKO;Tbx5het* mice. These cells were not associated with conducting airways, arteries, or veins and are inferred to be myofibroblasts. Similar ectopic interstitial ACTA2^+^ cells are seen in patients with TBX4 syndrome (Figure 5H). Full section confocal scans from each genotype are included for reference in Supplemental Figure 6.

### Pleural SMC bands are found with increasing density with T-box allele loss and are characterized by concurrent expression of myofibroblast and airway SMC markers.

**Figure 6:**
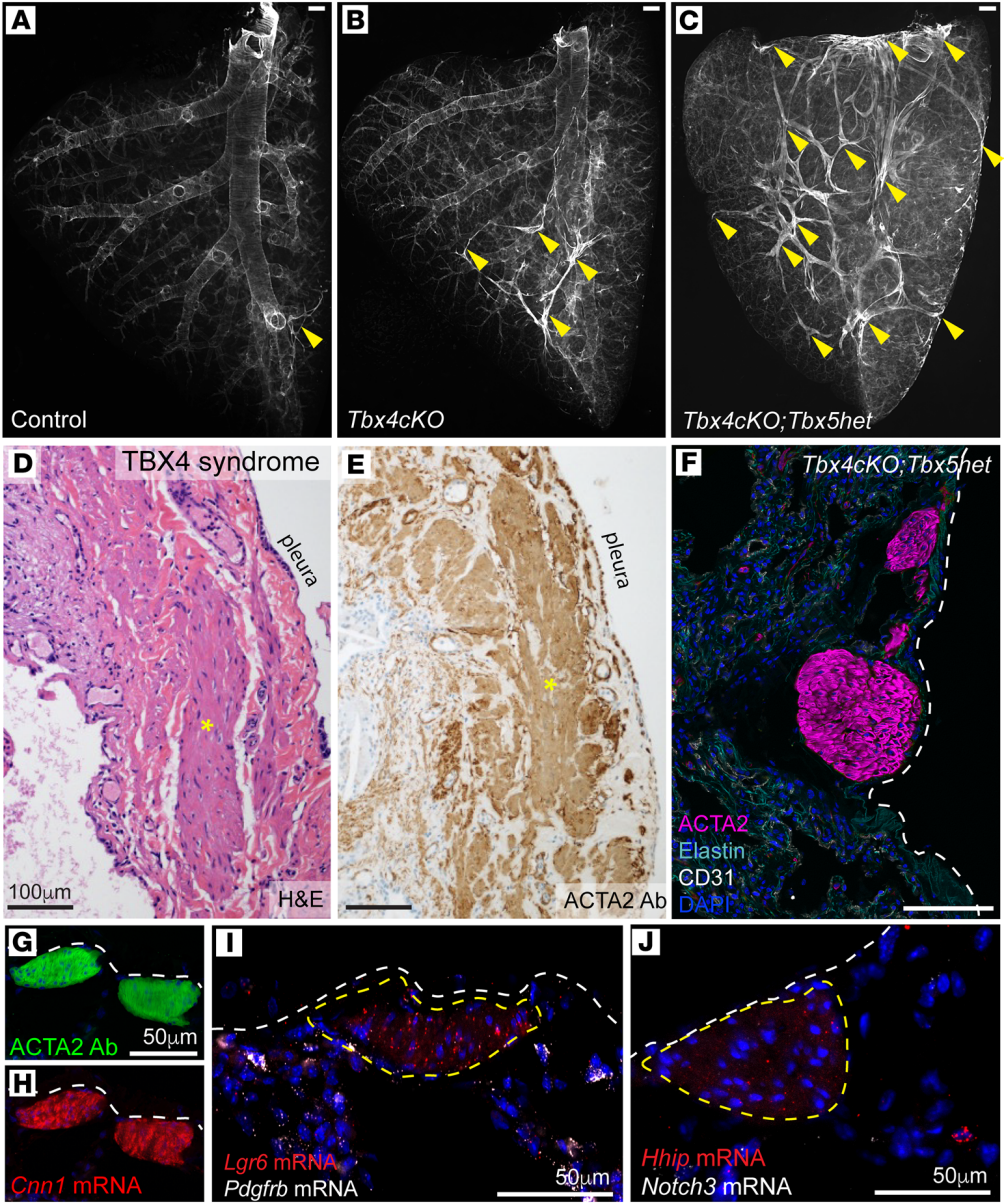
Loss of T-box genes leads to expansion of ectopic subpleural smooth muscle bands. (**A**–**C**) Ventral views of right caudal lobes from control, *Tbx4cKO*, and *Tbx4cKO;Tbx5het*, stained in whole mount to identify ACTA2^+^ cells (white). Small bands of subpleural smooth muscle (arrowheads) are occasionally found in control specimens, with the size and extent of the subpleural smooth muscle banding increasing in *Tbx4cKO*, and with *Tbx4cKO;Tbx5het* animals showing extensive banding wrapping all faces of the lobe. (**D** and **E**) Subpleural accumulation of ACTA2^+^ smooth muscle (asterisks) is a feature of TBX4 syndrome but absent in control (see Supplemental Figure 3). (**F**) Confocal micrograph of immunostained cryosection shows that mouse ACTA2^+^ bands lie directly beneath the pleura and are composed of bundles of tightly aligned cells that lack apparent contact with internal lung structures. Molecular characterization of ectopic subpleural bands in a *Tbx4cKO;Tbx5het* mouse by fluorescent in situ hybridization shows ACTA2^+^ bands express high levels of *Cnn1* (**G** and **H**) and *Lgr6* (**I**), minimal levels of *Hhip* (**J**), while *Notch3* and *Pdgfrb* are undetectable, not fully consistent with any canonical lung smooth muscle identity, but intermediate between airway smooth muscle and myofibroblast. Pleura are marked with a white dotted line in **F**–**J**; subpleural band cells are marked with a yellow dotted line in **I** and **J**. Scale bars: 500 μm (**A**–**C**), 100 μm (**D**–**F**), and 50 μm (**G**–**J**).

In addition to the airway and vascular abnormalities described above, whole-mount ACTA2 antibody staining of intact lobes revealed the presence of ACTA2-expressing bands, predominantly running along the ventral face of the pleural surface (Figure 6, A–C). These bands were composed of long strands of parallel smooth muscle–like fibers grouped in coherent bundles (Figure 6E). Short, thin bands were present in control specimens (Figure 6A). However, in *Tbx4cKO* and *Tbx4cKO;Tbx5het*, we observed a graduated increase in the length, density, and number of these pleural smooth muscle fibers (Figure 6, B and C). Although bands were prominent in all *Tbx4cKO;Tbx5het* lobes examined, in 1 animal elaborate webs of SMC bands covering the majority of the pleural surface were observed, to the point of obscuring visualization of underlying structures with fluorescence microscopy (Figure 6C). These bundles wrap around lobe edges and extend through clefts but never invade the lung parenchyma. Notably, they do not appear to connect to any internal patterning elements such as airways or vessels. This phenomenon of ectopic bundles of SMCs at the pleural surface has been previously reported in patients with TBX4 syndrome (Figure 6, D and E) (20) but their clinical consequence is poorly understood. To further characterize the molecular profile of pleural bands, we performed multiplexed RNA in situ hybridization using markers for mesenchymal subsets identified by recent single-cell profiling efforts (29), including airway and vascular SMCs, pericytes, and myofibroblasts (Figure 6, F–J). Our analysis revealed that these cells strongly express transcripts characteristic of generic SMC identity (*Cnn1*; Figure 6H) as well as *Lgr6* (Figure 6I), a marker of embryonic airway SMCs (25) and a lineage marker for septation myofibroblasts (37). We observed low levels of airway SMC marker *Hhip* (Figure 6J) and negligible expression of vascular SMC and pericyte markers *Pdgfrb* and *Notch3* (Figure 6, I and J). Collectively, this expression pattern reflects a generic ACTA2^+^ identity with some molecular features resembling airway SMCs and myofibroblasts, but not precisely matching any described SMC subset present in the healthy lung.

## Discussion

In this study, we developed a mouse model of *TBX4*-related lung disease that employs a lung-mesenchyme-specific deletion strategy to bypass the embryonic lethality of global loss of *Tbx4*, enabling investigation of the late embryonic and adult pulmonary consequences of TBX4 syndrome in vivo. In this model, we comprehensively characterized the changes to both the arterial network and ACTA2-expressing lineages of the lung that result from *Tbx4* loss of function. The model recapitulates key aspects of TBX4 syndrome, including echocardiographic evidence of PH and characteristic histopathologic findings in the pulmonary vasculature, airways, and pleura, with features worsening postnatally (9, 19–21). Given evidence of a progressive phenotype, this model offers an invaluable tool to study the mechanisms by which *TBX4*-related lung disease develops embryonically and worsens in postnatal life.

We provide several contributions to the understanding of *TBX4*-related lung disease. Combining tissue-specific mouse genetics, echocardiographic assessment, investigation of both late embryonic and adult time points, detailed microscopy, immunohistochemistry, and RNA in situ hybridization, we establish translational connections between this model and human TBX4 syndrome. Our 3-dimensional approach revealed complex structural changes that would be missed in traditional 2-dimensional analyses, including elaboration of the arterial branching network, abnormal distal muscularization of both arteries and airways, excessive parenchymal myofibroblasts, and the presence of ectopic subpleural smooth muscle bands, all features of TBX4 syndrome. Furthermore, this study represents the first comprehensive assessment to our knowledge of late embryonic PA and airway SMC distribution in homozygous *Tbx4* mutants, providing crucial insights into the developmental origins and postnatal disease progression in TBX4 syndrome.

The temporal aspects of our findings are particularly relevant to understanding human disease progression. At E18.5, we observe reduced lobe volumes and subtle but significant shortening of the distance between muscularized arteries and airways to the pleural edge. However, the diameter of vessels with complete SMC coat at the distal endpoint was unchanged between genotypes, contrasting with the extension of SMC coverage to smaller diameter arterioles and medial thickening observed in adults. These findings suggest that embryonic abnormalities may reflect hypoplastic lobes with likely epithelial underdevelopment and altered spatial patterning of smooth muscle, while progressive artery wall remodeling develops postnatally.

A recent study by Maldonado-Velez et al. (38) using a similar lung-mesenchyme-specific *Tbx4* deletion strategy demonstrated PH development with alveolar simplification, medial thickening, and what they interpreted as vascular simplification based on decreased von Willebrand factor–positive vessel density in 2D sections (38). Decreased vascular density was also reported in an early postnatal loss of *Tbx4* mouse model using similar methodology (39). However, our 3D whole-mount approach with elastin and ACTA2 staining reveals a more complex picture. Rather than vascular simplification, we observed an elaborate arterial bed with aberrant muscularization. Specifically, *Tbx4cKO* mice have distal muscularization of smaller diameter arteries in addition to more numerous muscularized arteries that become more pronounced with additional T-box gene loss. Although increased arterial branching might initially appear to reflect neovascularization with potential to reduce pulmonary vascular resistance, this finding occurs alongside distal muscularization of small diameter vessels, medial thickening, and luminal narrowing, likely accounting for echocardiography evidence of PH seen in this model. Together, these observations suggest a mechanism of increased SMC recruitment to arteries, which thickens the medial layer and aberrantly extends SMC coverage to the tiniest arterioles abutting the capillary network. These findings are consistent with intrinsic pulmonary arterial remodeling that, combined with the parenchymal abnormalities previously reported (38) and observed but not specifically interrogated in this work, likely contributes to PH pathogenesis in TBX4 syndrome. The apparent discrepancy with the previously published findings (38, 39) likely reflects methodological differences; 2D vessel density measurements can be influenced by the significant alveolar enlargement they reported, where increased airspace size may reduce apparent vessel density per high-power field even without true vessel loss. Our 3-dimensional approach, which quantifies arterial architecture along defined anatomical branches independently of airspace size, reveals the underlying vessel elaboration.

The smooth muscle wrapping airways is also abnormal, with widened spacing of smooth muscle rings and extension of sparse ACTA2^+^ bands into the distal lung parenchyma, reaching toward the pleural surface and running with the arteries. Experiments to delineate the border between the conducting and respiratory epithelium in these altered airways, and identify how these changes relate to the alveolarization defect described by Maldonado-Velez et al., are needed (38). The pathophysiologic consequences of the airway-associated smooth muscle changes we observe are unknown, although muscularized conducting airways close to the pleura have been described in TBX4 syndrome (20). Pulmonary function testing in adult *Tbx4cKO* mice represents an important direction for future investigation. The postnatal TBX4 deletion model reported increased lung resistance and decreased compliance (39), findings that may be related to the airway smooth muscle abnormalities described here, although whether similar airway smooth muscle changes are present in that model or similar pulmonary function consequences exist in ours remains to be established.

We observed C-shaped ACTA2^+^ cells consistent with myofibroblasts in the parenchyma of *Tbx4cKO* lungs (37). These myofibroblasts were absent in controls and more prominent in *Tbx4cKO;Tbx5het* mice, and ectopic interstitial ACTA2^+^ cells are found in patients with TBX4 syndrome. Their distribution resembles the pattern of airway-associated ACTA2^+^ “septation myofibroblasts” present during alveologenesis (postnatal days 4–30). These cells originate as tightly parallel Lgr6^+^ SMC bundles wrapping embryonic airways, which then separate and rearrange to enable epithelial budding and the formation of alveoli (37). Septation myofibroblasts downregulate ACTA2 expression by postnatal day 30, and are not visualized by ACTA2 staining of adult controls (37). The distribution of ACTA2^+^ myofibroblasts in adult *Tbx4cKO* and *Tbx4cKO;Tbx5het* mice mimics that of septation myofibroblasts present during early postnatal life. This suggests *Tbx4* and *Tbx5* are critical for the appropriate suppression of myofibroblast smooth muscle gene expression at the completion of alveologenesis.

A further example of abnormal ACTA2^+^ cell distribution is the appearance of elaborate subpleural bands in the *Tbx4cKO* and *Tbx4cKO;Tbx5het* lungs. The molecular characterization of these subpleural structures revealed they do not correspond to any SMC subtype normally present in healthy lung tissue, although they exhibit more myofibroblast and airway smooth muscle–like features than markers typical of vascular SMCs and pericytes (29). These bands represent another manifestation of a broad dysregulation of mesenchymal cell fate and a propensity among mesenchymal cells to differentiate to an SMC identity with loss of *Tbx* gene function. The precise mechanisms triggering subpleural band development, the molecular signals controlling their expansion, and the precise identity of their cell of origin need to be defined in the future. Regarding functional consequences, while little is known about the physiologic impact of subpleural SMC bands, we hypothesize they could restrict lung expansion in a manner analogous to fibrotic pleural disease, although the mechanism is distinct given that these are SMC bundles rather than fibrotic tissue. Notably, ectopic subpleural smooth muscle accumulation has been reported in other genetic mouse models of lung mesenchymal dysregulation, including *Myrf* and *Ezh2* mutants, suggesting a shared phenotypic consequence of disrupted mesenchymal fate regulation in the developing lung (40, 41). The clinical relevance of these bands in TBX4 syndrome and their potential contribution to patients’ respiratory limitations warrants future investigation.

The pathologic muscularization of the pulmonary arteries, altered patterning of airway smooth muscle, persistence of ACTA2^+^ myofibroblasts within the interstitium, and ectopic subpleural smooth muscle bands each represent examples of dysregulated smooth muscle acquisition or patterning driven by the loss of *Tbx4*. These together point to *Tbx4* as a key regulator of smooth muscle differentiation whose loss leads to overabundance of ACTA2^+^ cells in multiple lung compartments. The mechanism by which this SMC phenotype is acquired appears to differ among compartments, and future studies interrogating shared and divergent downstream signaling networks in each distinct mesenchymal subset in this model are needed.

The combination of arterial remodeling, airway smooth muscle abnormalities, and parenchymal myofibroblasts observed in our model, along with previously reported alveolar simplification (38, 39), reflects the complex pathophysiology of TBX4 syndrome (9, 19, 20). Clinically, *TBX4*-related PH can be classified within World Health Organization Group 1 (PAH) or Group 3 (PH associated with lung diseases) (16). Findings from this study support this classification heterogeneity; while primary arterial remodeling with pathologic muscularization characteristic of PAH is seen, there are concurrent airway abnormalities and persistence of ACTA2^+^ myofibroblasts within the interstitial space, suggesting that interstitial lung disease may also be contributing to PH pathogenesis in these animals.

This study has several limitations as a model of TBX4 syndrome. First, by design, we are investigating a pure lung phenotype that lacks compounding effects from changes to other organs, particularly the allantois and placenta, and therefore we have necessarily lost any comorbid contributions that may worsen lung disease in humans (42). Beyond the genetic approach of the model, there are several methodological limitations. Our study is primarily descriptive, leaving the molecular mechanisms underlying aberrant smooth muscle differentiation undefined. Although TBX4 is exclusively expressed in lung mesenchyme, the pulmonary vascular remodeling and alveolar simplification observed in both this model (38) and human TBX4 syndrome demonstrate that loss of TBX4 in the mesenchyme has secondary effects on endothelial and epithelial compartments, likely through disrupted cell-cell signaling. The quantitative extent of these compartment-specific changes and the molecular mechanisms mediating this mesenchymal-to-endothelial and mesenchymal-to-epithelial crosstalk remain incompletely characterized and represent important directions for future investigation. In addition, animals were collected between 3 and 9 months of age, which is a wide age range for a model in which we see disease progression. Notably, while controls and *Tbx4cKO* mice had an even age distribution, *Tbx4cKO;Tbx5het* animals skewed younger within this range, such that any age-related contribution to vascular remodeling would bias against detecting more severe phenotypes in this genotype, suggesting our observed differences represent a conservative estimate of the true genotype effect. Finally, although RV catheterization is considered the gold standard for PH diagnosis in mice, invasive hemodynamic confirmation has been established in a parallel lung mesenchyme-specific *Tbx4* deletion model (38) that exhibited statistically significant increases in RV systolic pressure and RV hypertrophy by the Fulton index. Therefore, we used echocardiography as a non-invasive approach to infer evidence of PH in this paper. We acknowledge that echocardiography is an indirect measure of PH and the lack of direct hemodynamic confirmation remains a limitation of the current study.

Potential compensatory or redundant functions of other pulmonary-expressed T-box transcription factors in TBX4-deficient mesenchymal cells have not been systematically assessed. Prior work has demonstrated that Tbx4 and Tbx5 interact in the developing respiratory system and that loss of *Tbx5* worsens the lung phenotype in the context of *Tbx4* loss of function (22). The *Tbx4cKO;Tbx5het* experiments therefore leverage readily available genetic tools to test the phenotypic consequences of reduced T-box dosage in the lung mesenchyme. A large portion of TBX4 syndrome is secondary to a microdeletion that includes both *TBX4* and its homolog *TBX2*. Like *TBX4*, both *TBX2* and *TBX5* are expressed in the lung mesenchyme (24, 25, 28, 29) and some level of functional redundancy between the homologs is expected, but the field would benefit from a clinical relevant model of the 17q23 microdeletion containing *TBX4* and *TBX2*. Given the phenotypic heterogeneity characteristic of haploinsufficient heritable pulmonary vascular disease including TBX4 syndrome, future studies incorporating environmental second-hit insults such as hypoxia, hyperoxia, or inhalation injury could leverage this model to interrogate genotype-specific responses to these exposures. Notably, cellular origins of the aberrant ACTA2^+^ subsets described in this model, including the arterial and airway associated expansions as well as the parenchymal myofibroblast and subpleural populations, have not been formally established through lineage tracing. Identifying the progenitor cell types driving each of these compartment-specific changes represents an important and meaningful direction for future investigation.

In conclusion, our findings reveal that TBX4 functions as an important regulator of smooth muscle differentiation within the pulmonary mesenchyme, with its loss driving excessive smooth muscle acquisition and altered patterning across both airway and vascular compartments, underscoring its crucial role in normal lung development and homeostasis. This model reveals a phenotype that closely recapitulates the clinical pulmonary spectrum of TBX4 syndrome in humans, with embryonic origins that progressively worsen postnatally (20, 21). Three-dimensional whole-mount analysis and high-resolution spatial quantitation uncovered complex structural changes that would remain hidden in traditional 2-dimensional histologic approaches. This high-resolution spatial approach allowed us to show that *Tbx4* loss results not in vascular simplification, but rather in an abnormally elaborated and over-muscularized pulmonary arterial tree. Importantly, these data suggest that *TBX4*-related pulmonary vascular disease may represent not merely a fixed developmental defect but rather a progressive postnatal pathologic process dominated by ongoing mesenchymal dysregulation and SMC acquisition. This suggests a possible therapeutic window when pharmacologic inhibition of aberrant smooth muscle differentiation and proliferation pathways could be targeted and may be targeted to attenuate PH progression, potentially reducing patient morbidity and mortality.

## Methods

### Sex as a biological variable.

Both male and female mice were evaluated in this study, with no overt histologic phenotypic differences observed between sexes. Echocardiographic assessment was completed on male and female mice across genotypes. The study was not specifically powered to detect sex-based differences in *TBX4*-related pathology.

### Mice.

The *Tbx4LMECre*, *Tbx4* conditional (floxed) allele, and *Tbx5* conditional allele mice have been previously described (26, 30, 31). *Tbx4^fl/+^* mice (stock Tbx4tm1.2Pa/Mmjax, RRID:MMRRC_043812-JAX), were obtained from the Mutant Mouse Resource and Research Center (MMRRC) at The Jackson Laboratory, an NIH-funded strain repository, and were donated to the MMRRC by Virginia Papaioannou (Columbia University Medical Center, New York City, New York, USA). *Tbx5^fl/+^* mice were provided by Benoit Bruneau (Gladstone Institute, San Francisco, California, USA). *Tbx4LMECre; Tbx4^fl/fl^* (herein referred to as *Tbx4cKO*) mice were made by mating *Tbx4LMECre* males with conditional allele *Tbx4^fl/fl^* females and subsequently crossing *Tbx4LMECre; Tbx4^fl/+^* males with *Tbx4^fl/fl^* females to reach homozygosity of *Tbx4* conditional alleles. Animals for analysis were generated by crossing *Tbx4cKO* with *Tbx4^fl/fl^*, and Cre-negative littermates served as controls. Similarly, *Tbx4LMECre; Tbx4^fl/fl^; Tbx5^fl/+^* (herein referred to as *Tbx4cKO;Tbx5het*) mice were made by mating *Tbx4cKO* males with *Tbx4^fl/fl^*; *Tbx5^fl/fl^* females, resulting in homozygous *Tbx4* conditional alleles and heterozygous *Tbx5* conditional and wild-type alleles. Gestational age was determined by observation of a vaginal plug (designated as day 0.5).

### Genotyping and primers.

Excision of *Tbx4^fl^* was analyzed by PCR. Embryonic lungs were dissected at E18.5, and hearts and tracheas were removed. Embryonic lung tissue was lysed in lysis buffer (3% proteinase K) overnight at 55°C, followed by enzyme inactivation for 10 minutes at 95°C. Lysed lung tissue was genotyped for Cre and *Tbx4* as previously described (22). The full list of PCR primers used is listed in Supplemental Figure 1C. The conditional allele was effectively excised in Cre-positive lungs, as indicated by the decrease in intensity of the conditional band and presence of the null band (Supplemental Figure 1A). PCR amplification was performed using a Bio-Rad T100 Thermal Cycler. The cycling conditions for *Tbx4* were initial denaturation at 95°C for 2 minutes, followed by 40 cycles of denaturation at 95°C for 30 seconds, annealing at 63°C for 30 seconds, extension at 72°C for 30 seconds, and a final extension at 72°C for 5 minutes, with a final hold at 4°C. The cycling conditions for Cre were initial denaturation at 94°C for 3 minutes, followed by 40 cycles of denaturation at 94°C for 30 seconds, annealing at 60°C for 30 seconds, extension at 72°C for 30 seconds, and a final extension at 72°C for 10 minutes, with a final hold at 4°C.

### PH assessment by echocardiography.

Cardiac ultrasound imaging (echocardiography) was performed on adult mice. Mouse genotypes were deidentified to ensure experimental blinding. Mice were anesthetized using 4% isoflurane in oxygen at 1 L/min in a chamber, and anesthesia was maintained using 1.5%–2% isoflurane in oxygen at 0.6 L/min delivered through a nose cone for the duration of imaging. Animals were kept on a heated platform while under anesthesia and heart rate was monitored using electrocardiogram leads. Fur was removed from the thorax using electric clippers and chemical depilatory immediately prior to imaging. A VevoF2 (Fujifilm VisualSonics) high-frequency ultrasound with a UHF57x (25–57 MHz) transducer was used to image the hearts and great vessels in short axis and long axis parasternal views. The Doppler function was used to assess cardiac blood flow (43).

PAT and PET were measured in PW Doppler Mode across 3 cardiac cycles. The averages of these 3 measurements were used to calculate PAT/PET. RVOT was measured from a parasternal long axis view using B-Mode. RV and LV areas were measured from short axis B-Mode images in systole and diastole. CO, SV, and EF were measured from a parasternal long axis view.

### Embryonic tissue collection.

Embryonic lungs were collected from timed matings at E18.5. Pregnant mice were euthanized at E18.5 using carbon dioxide asphyxiation and cervical dislocation. Embryos were dissected from the uterus with yolk sac intact and immediately placed on ice. Lungs were dissected from embryos in ice-cold 0.1% BSA in PBS. Lung lobes were separated, fixed in DMSO/methanol (1:4), and stored at –20°C overnight.

### Adult tissue collection, perfusion, inflation, fixation, and storage.

Adult lungs were collected from age-matched littermate *Tbx4cKO* or *Tbx4cKO;Tbx5het* mice and controls between 4 and 9 months of age. Mice were euthanized using carbon dioxide asphyxiation and lungs were perfused using approximately 30 mL of ice-cold PBS via RV puncture. Lungs were inflated using 2% low-melting-point agarose in PBS, placed on ice, and lung lobes were separated and fixed individually. Caudal lobes were fixed in 4% paraformaldehyde (PFA) for 4 hours followed by serial dehydration in 3:1, 1:1, and 1:3 solutions of PBS/methanol, then placed in 100% methanol and stored at –20°C until ready for staining. Left lungs were fixed in 4% PFA for 4 hours followed by serial dehydration in 10%, 20%, and 30% solutions of sucrose in PBS, then embedded in OCT and stored at –80°C until ready for staining.

### Histology and immunohistochemistry.

Cryosections were prepared and stained following standard protocols (44). Briefly, 4% PFA–fixed lobes were cryopreserved in 30% sucrose in PBS, embedded in OCT (Sakura, 4583), and 20-μm sections were cut using a Leica CM3050S cryostat. Cut sections were stored at –80°C prior to staining. On the day of staining, slides were thawed, washed in PBS with 0.1% Tween 20 (PBT) twice for 10 minutes, blocked at least 30 minutes in preblock consisting of 0.3% Triton X-100, 5% serum, and 1.5% BSA, and then incubated overnight in primary antibody solution diluted in preblock at room temperature. The next day, slides were washed (twice for 10 minutes each with PBT), incubated 45 minutes in secondary antibody solution, nuclei were stained with DAPI (1:1000 dilution of 10 mg/mL stock; Invitrogen, D1306), and slides were mounted with Prolong Gold antifade mounting media (Thermo Fisher Scientific, P36930). For visualization of elastin fibers by staining with fluorescent hydrazide dye (45), stock solutions were prepared by dissolving 1 mg hydrazide-A633 (Invitrogen, A30634) in 2 mL diH_2_O, and were used at a 1:500 dilution in the primary or secondary antibody mix.

Primary antibodies used were mouse anti-ACTA2-Cy3 (Sigma-Aldrich, C6198, clone 1A4; 1:200), mouse anti-ACTA2-FITC (Sigma-Aldrich, F3777, clone 1A4; 1:200), rat anti-mouse CD31 (BD Pharmingen, 553370, clone MEC 13.3; 1:500), and hamster anti-mouse CD31 (Bio-Rad, MCA1370Z, clone 2H8; 1:200). Secondary antibodies used were Alexa Fluor 647–conjugated goat anti-hamster (Invitrogen, A-21451; 1:250) and Alexa Fluor 488–conjugated goat anti-rat (Invitrogen, A-11006; 1:250).

### Whole-mount antibody staining.

Whole-mount immunofluorescent staining of E18.5 lungs was performed as previously described (46). Embryonic lung lobes fixed in DMSO/methanol were rehydrated in 1:1 methanol/PBS with 1% Triton (PBS-Triton) for 15 minutes, then washed in PBS-Triton for 15 minutes at room temperature. Lung lobes were then incubated in 2% powdered milk in PBS-Triton blocking solution for 2 hours. Blocking solution was removed and primary antibodies were added (1:250 antibody dilution in blocking solution). Lung lobes were incubated in primary antibody, mouse anti-ACTA2-FITC (Sigma-Aldrich, F3777; 1:250), for 5 nights at 4°C. Lung lobes were then washed in PBS-Triton twice for 15 minutes and 4 times for 45 minutes each at room temperature. Hoechst (B2261, Sigma-Aldrich; 1:250) was diluted in PBS-Triton and lung lobes were incubated for 3 nights at 4°C. Next, lung lobes were washed in PBS-Triton twice for 15 minutes and 4 times for 45 minutes each at room temperature, then dehydrated in methanol/PBS-Triton (1:1) followed by 100% methanol for 30 minutes. Lung lobes were subsequently bleached in 3% hydrogen peroxide in methanol at 4°C overnight. Lastly, bleached samples were washed in 100% methanol for 30 minutes and cleared overnight in benzyl alcohol/benzyl benzoate (1:2) (BABB).

Adult lung lobes stored in 100% methanol were rehydrated, bleached in 5% hydrogen peroxide in PBS, washed in PBS-Triton (twice, 10 minutes each), incubated with preblock consisting of 0.3% Triton X-100, 5% serum, and 1.5% BSA for 1 hour, and then incubated for 5 nights in primary antibody solution containing anti-ACTA2-Cy3 (Sigma-Aldrich, C6198; 1:200) and hydrazide-A633 (Invitrogen, A30634; 1:500 dilution of 0.5 mg/mL stock) diluted in preblock at 4°C. Samples were then washed (PBS-Triton twice, 10 minutes each), postfixed briefly in 4% PFA (20 minutes), dehydrated to 100% methanol in glass container (30 minutes 50% methanol, 30 minutes 75% methanol, 30 minutes 100% methanol, 30 minutes 100% methanol) and cleared in BABB for 20 minutes and then stored in BABB at 4°C.

### Confocal imaging and quantitation.

Embryonic lung samples were imaged using a Leica TCS SP8 X confocal laser scanning microscope system with white-light laser and 20× BABB immersion objective. The full volume of the lung lobes was imaged using tile scans with a *z*-step size of 7 μm. Tile scan images were merged using Leica software LASX version 3.5.5 (47). Image analysis for embryonic lung lobes was performed using Imaris v9.2.1 by importing confocal image (.LIF) files into Imaris Surpass mode. PA and airway SMC metrics were analyzed by creating a surface for smooth muscle and the pleural edge. Caudal lobe volume was analyzed by creating a surface of Hoechst staining and using surface statistics in Imaris.

All adult immunohistochemistry and RNAscope (see *RNA in situ hybridization* below) images were captured on a Zeiss 880 Examiner confocal microscope 20× or 40× objective with glycerol and minimally processed with Zen software (Carl Zeiss AG).

### PA morphology assessment from cryosection stains.

Media thickness and percentage artery diameter for neointima were calculated from single-plane confocal images as follows. Cryosections (20 μm) were prepared from lungs of all 3 genotypes and stained with hydrazide to mark elastic laminae, ACTA2 to mark SMCs and neointima, CD31 to mark endothelium, and DAPI to mark nuclei. Three to 5 stained sections were imaged at 20× by tiled confocal microscopy, and all PA cross sections with diameters between 20 μm and 200 μm were identified for measurement in the stitched files. For each PA, the following measurements were taken manually using Zen Black (Carl Zeiss AG) software: external vessel diameter (measuring from outside margin of external elastic laminae), media thickness (inside margin of external elastic lamina to outside margin of internal elastic lamina), and neointima thickness (inside margin of internal elastic lamina to the outside edge of the endothelium). Two sets of orthogonal measurements were taken for each PA and averaged. Percentage lumen was calculated as ([vessel diameter – media thickness – neointima thickness]/vessel diameter) × 100. Three animals were analyzed for each genotype and for each animal 25–50 arteries were measured. Vessels in cross section were included for morphometric analysis, as oblique cuts may overestimate medial thickness and underestimate lumen diameter; this exclusion criterion was applied uniformly across genotypes. Genotypes were deidentified and blinded prior to quantitation.

### Stereoscope imaging and quantitation.

For adult whole-lobe analysis and detailed analysis of SMC and elastin distribution along peripheral arteries and airways, multi-focus stereoscopic images at 1× and 2× magnification of the specified branches were collected using an M205 FA fluorescent stereoscope (Leica Microsystems), Orca-Flash 4.0 LT monochrome digital camera (Hamamatsu), and LASX software (Leica Microsystems). Measurements were made using Fiji, a distribution of ImageJ (NIH). Branches that were selected for analysis are ones that are arrayed in a single flat plane, such that measurements from multi-focus images accurately capture the distance to pleura.

### Acquisition, processing, and staining of human tissue.

Lung tissues were obtained at autopsy, fixed in 10% neutral buffered formalin and processed, embedded, sectioned, and H&E stained following standard protocols (21). ACTA2 immunostaining was done via automated staining using a Ventana BenchMark XT automated slide stainer (Leica Microsystems).

### RNA in situ hybridization.

Fluorescent in situ hybridization on 20-μm cryosections sections was carried out using an RNAscope v2 kit (ACDbio, 323100), following the manufacturer’s instructions. RNA in situ hybridization protocol was followed by a 5-minute DAPI stain and overnight incubation with anti-ACTA2-FITC antibody (Sigma-Aldrich, F3777) diluted 1:200 in preblock solution. Mouse probes used were 483791 (*Cnn1*), 425171 (*Notch3*), 411381 (*Pdgfrb*), 404961 (*Lgr6*), and 448441 (*Hhip*), all from ACDbio.

### Statistics.

Statistical analysis for medial and neointimal thickness, PA and airway distance to the pleura, percentage lumen, artery SMC coverage length, distal PA diameter, and airway SMC ring gapping was performed using Welch’s ANOVA (assuming unequal variances) to assess differences across genotypes, followed by Games-Howell post hoc tests for pairwise comparisons between groups. For lobe size, mouse weights and echocardiographic parameters statistical analysis was performed using 1-way ANOVA across genotypes, followed by Tukey’s HSD post hoc tests for pairwise comparisons between groups. A *P* value of less than 0.05 was considered significant. Data are presented as box-and-whisker plots (line, median; box bounds, IQR; whiskers, 1.5 × IQR) with individual data points overlaid. All statistical analysis and plotting were done in the statistical language R (www.R-project.org) using RStudio (RStudio Inc). Plots were made using the ggplot2 R package (ggplot2.tidyverse.org).

### Study approval.

All animal maintenance and procedures were performed at Michigan State University in accordance with the Institutional Animal Care and Use Committee guidelines. Studies on human tissue were performed on deidentified single autopsy samples; therefore, Institutional Review Board (IRB) approval was deemed not required.

### Data availability.

The Supporting Data Values file contains the complete collection of raw measurements utilized throughout this investigation, providing the underlying numerical data for all figures and charts.

## Author contributions

LCS and KAC are co–first authors: LCS participated in study design, developed quantitative protocols, performed imaging and quantitation of adult lung samples, conducted all data analysis and visualization, imaged and analyzed RNAscope results, created figures, and wrote and revised the manuscript. KAC generated and characterized transgenic mouse lines, performed PCR validation, coordinated echocardiography of adult mice, harvested and fixed embryonic and adult mouse lungs, conducted embryonic staining, imaging, and quantitation, drafted text of embryonic methods and results sections, and critically revised the manuscript. The order of co–first authors was determined based on LCS’s primary role in data analysis, figure generation, and drafting initial manuscript, while KAC led the mouse tissue generation and handling, echocardiographic assessments, and embryonic analysis aspects of the study. SRM processed tissues, performed staining of adult mouse cryosections and whole-lobe preparations, conducted RNAscope protocols, and critically reviewed the manuscript. AR assisted with tissue processing and staining, performed quantitative vessel remodeling measurements, and critically reviewed the manuscript. MD generated transgenic mouse lines and critically reviewed the manuscript. CG provided expertise in human pathophysiology, contributed human histology examples, assisted with study design, and critically reviewed the manuscript. MEK and RA are co–corresponding authors: MEK designed the study, formulated research goals, developed histologic visualization and quantitation methods, mentored LCS, SRM, and AR, provided reagents for adult antibody staining and RNAscope, performed imaging and quantitation of adult lung samples, created and edited figures, drafted and critically revised the manuscript. RA developed the mouse model approach, funded mouse colony and echocardiography analysis, designed the study, formulated research goals, provided reagents for embryonic antibody staining, mentored KAC and MD, and critically revised the manuscript.

## Conflict of interest

The authors have declared that no conflict of interest exists.

## Funding support

This work is the result of NIH funding, in whole or in part, and is subject to the NIH Public Access Policy. Through acceptance of this federal funding, the NIH has been given a right to make the work publicly available in PubMed Central.

- Jean P. Schultz Biomedical Research Endowment Fund from College of Human Medicine, Michigan State University (to RA).
- Pediatric PH research award from the Pulmonary Hypertension Association (to RA).
- Eunice Kennedy Shriver National Institute of Child Health and Human Development/NIH award T32HD087166 (to KAC).
- NIH grant R01 HL163013-01 (to MEK).
- Esther Ehrman Lazard faculty scholarship endowment through the Department of Pediatrics at Stanford University (to MEK).
- NIH grant K08 HL173632 (to LCS).
- American Thoracic Society Early Career Investigator in Pulmonary Vascular Biology Award (to LCS).
- Instructor K Award Support through the Maternal and Child Health Research Institute at Stanford University Department of Pediatrics (to LCS).

## Supplementary Material

Supplemental data

Unedited blot and gel images

Supplemental video 1

Supplemental video 2

Supplemental video 3

Supporting data values

## Version 1 04/27/2026

In-Press Preview

## Version 2 06/08/2026

Electronic publication
