# Supplementary material for: Excessive postnatal smooth muscle differentiation in a lung-specific model of TBX4-related pulmonary hypertension
Source: JCI Insight. 2026 Apr 23;11(11):e194251. doi: 10.1172/jci.insight.194251 (PMC13313494; doi:10.1172/jci.insight.194251)
Supplement: Supplemental data [file jciinsight-11-194251-s207.pdf]

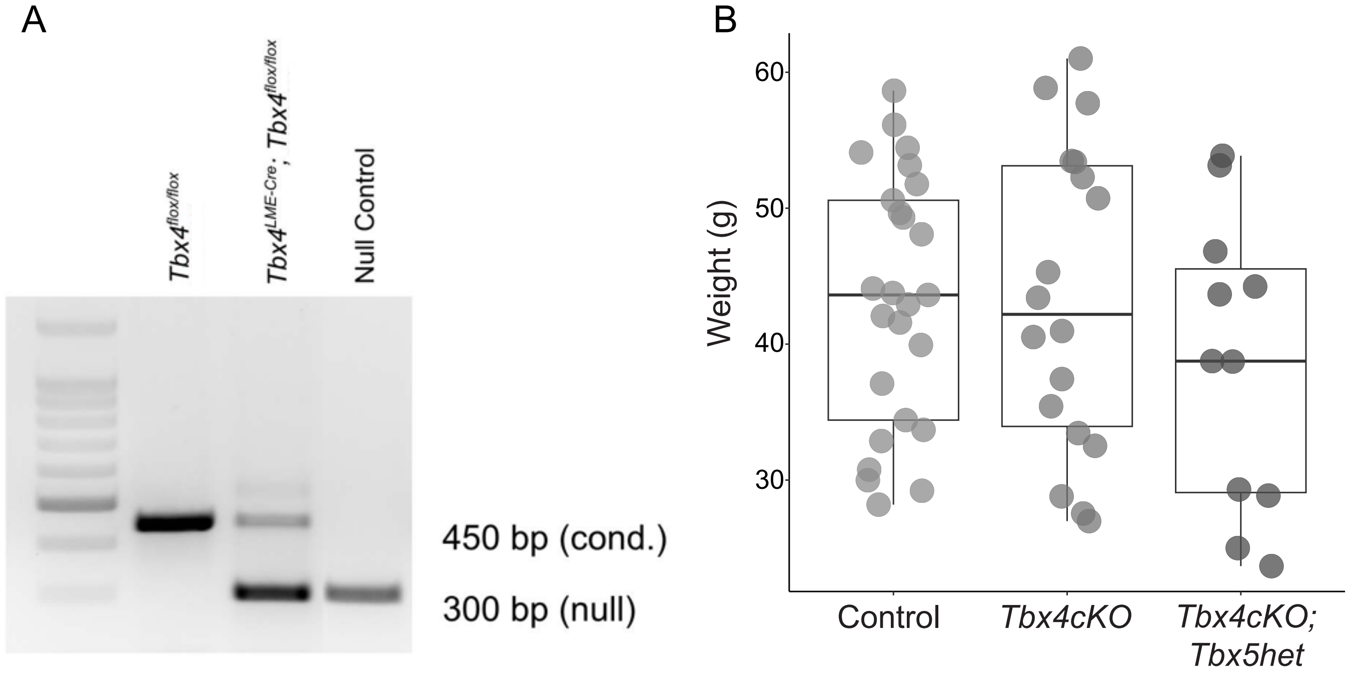

C

PCR primers used to genotype and assess conditional allele excision.

Primer	Sequence
Tbx4 Conditional Allele Excision	
Tbx4-F	GCAGATGACCATCGCTACAA
Tbx4-M	TCATCTAGGCTTCACAGCC
Tbx4-R	GCCAGGCAGAGAAACAAAAG

Supplemental Figure 1. Characterization of Tbx4 deletion in embryonic and postnatal mice. A, Semi-quantitative PCR was performed using three primers targeting the *Tbx4* conditional (“floxed”) and excised conditional (“null”) alleles. The conditional allele was effectively excised in Cre-positive lungs, as indicated by the marked decrease in intensity of the conditional band and presence of the null band. Non-mesenchymal cells carry floxed alleles without Cre expression; therefore, the presence of a conditional band is expected in both *Tbx4cKO* and conditional samples. As a control, the null band was generated using only the two primers targeting the null allele in a *Tbx4cKO* sample. **B**, No significant difference in body weight was found between control, *Tbx4cKO,* and *Tbx4cKO;Tbx5het* adult mice at the time of harvest. **C**, PCR primer sequences (Tbx4-F, Tbx4-M, and Tbx4-R) used for genotyping and verification of conditional *Tbx4* allele excision.

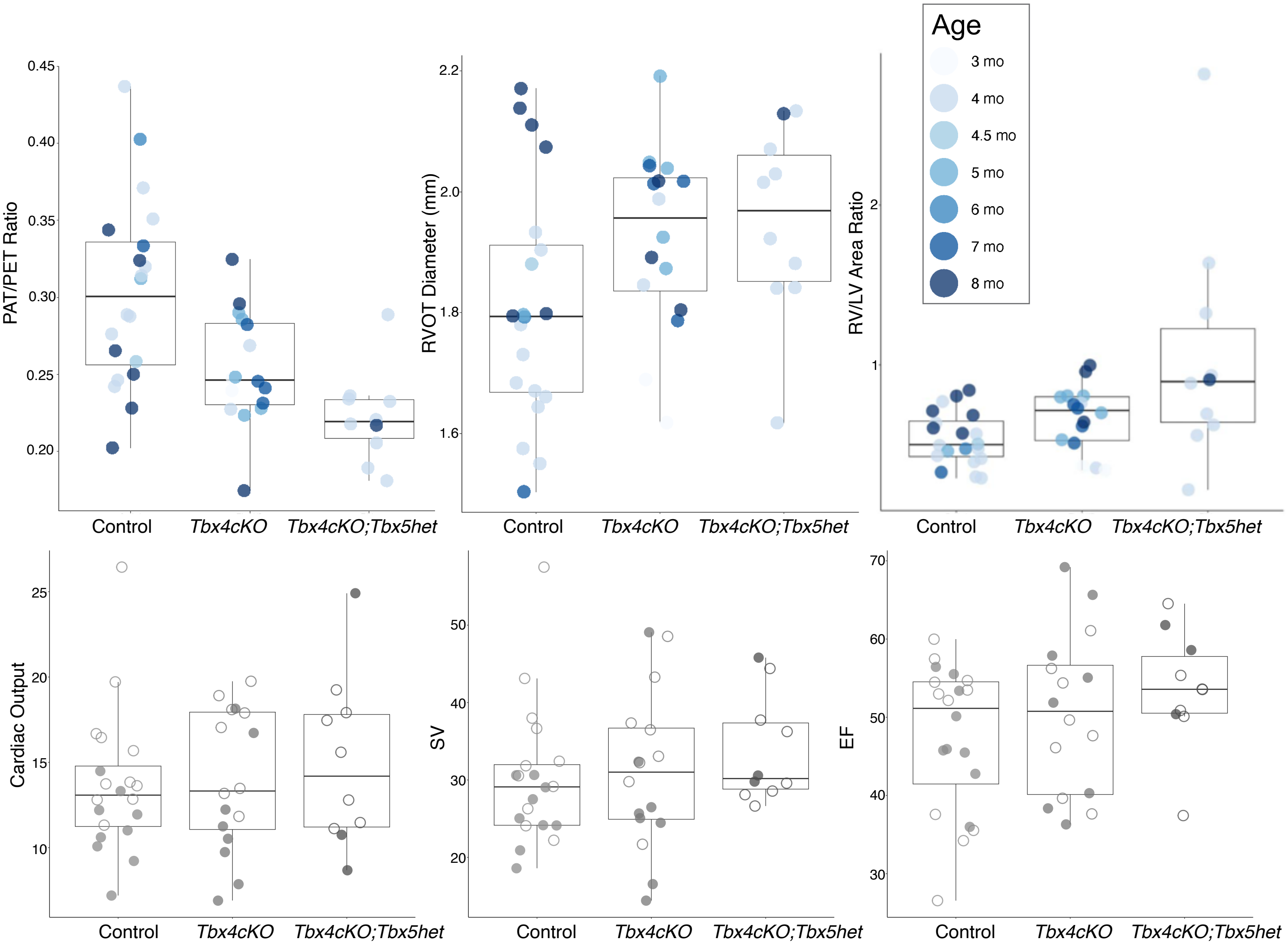

Supplemental Figure 2. Age distribution of right heart measurements and preserved left heart function across genotypes in *Tbx4cKO* and *Tbx4cKO;Tbx5het mice*. Top) PAT/PET ratio, RVOT diameter, and RV/LV area ratio across genotypes, with individual data points color coded by age at measurement. (Bottom) No significant difference in cardiac output (CO), stroke volume (SV), or ejection fraction (EF) between control, *Tbx4cKO*, and *Tbx4cKO;Tbx5het* mice. Statistical comparisons were performed using one-way ANOVA with Tukey's HSD post hoc testing. Males, open circles; females, closed circles.

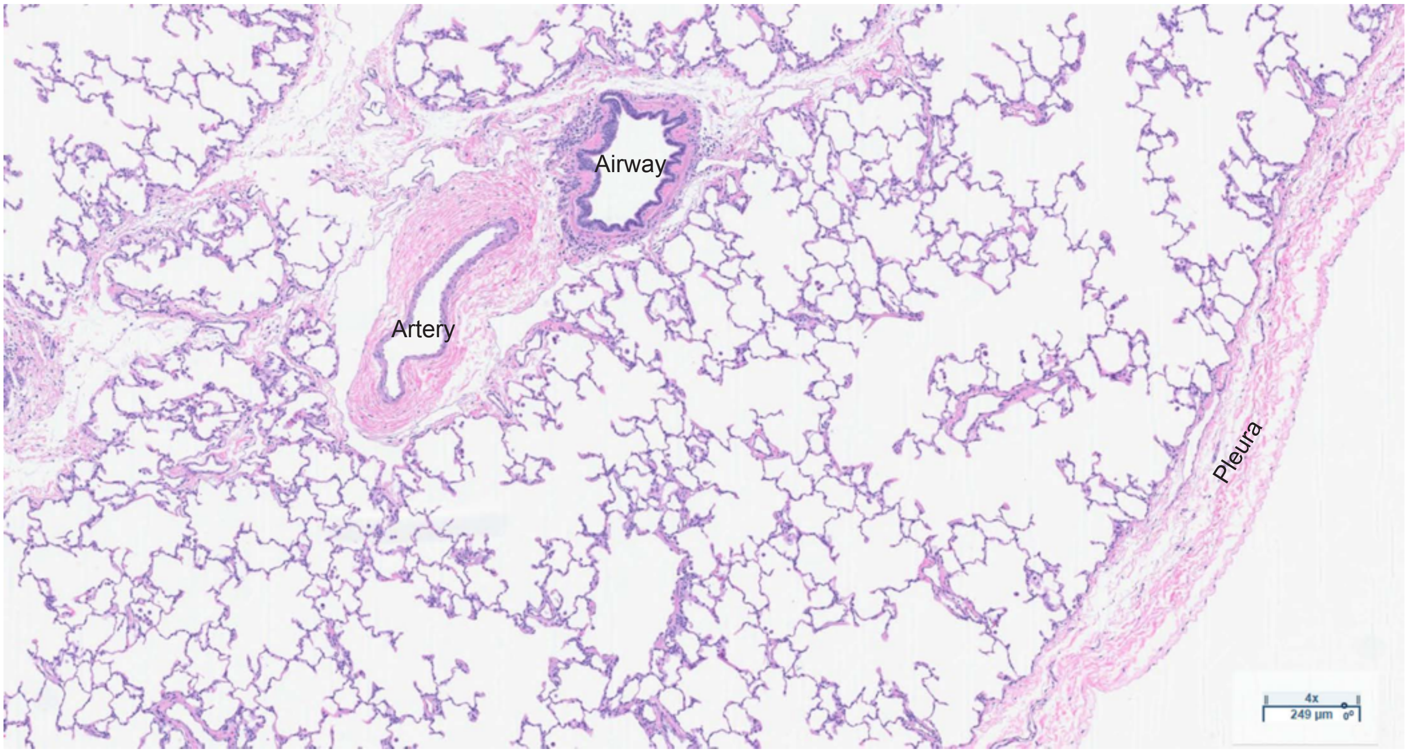

Supplemental Figure 3: Histology of normal human lung. Normal human lung histology demonstrating substantial space between the pleural edge and muscularized airways and arteries. H&E stain of parafin embedded control lung specimen.

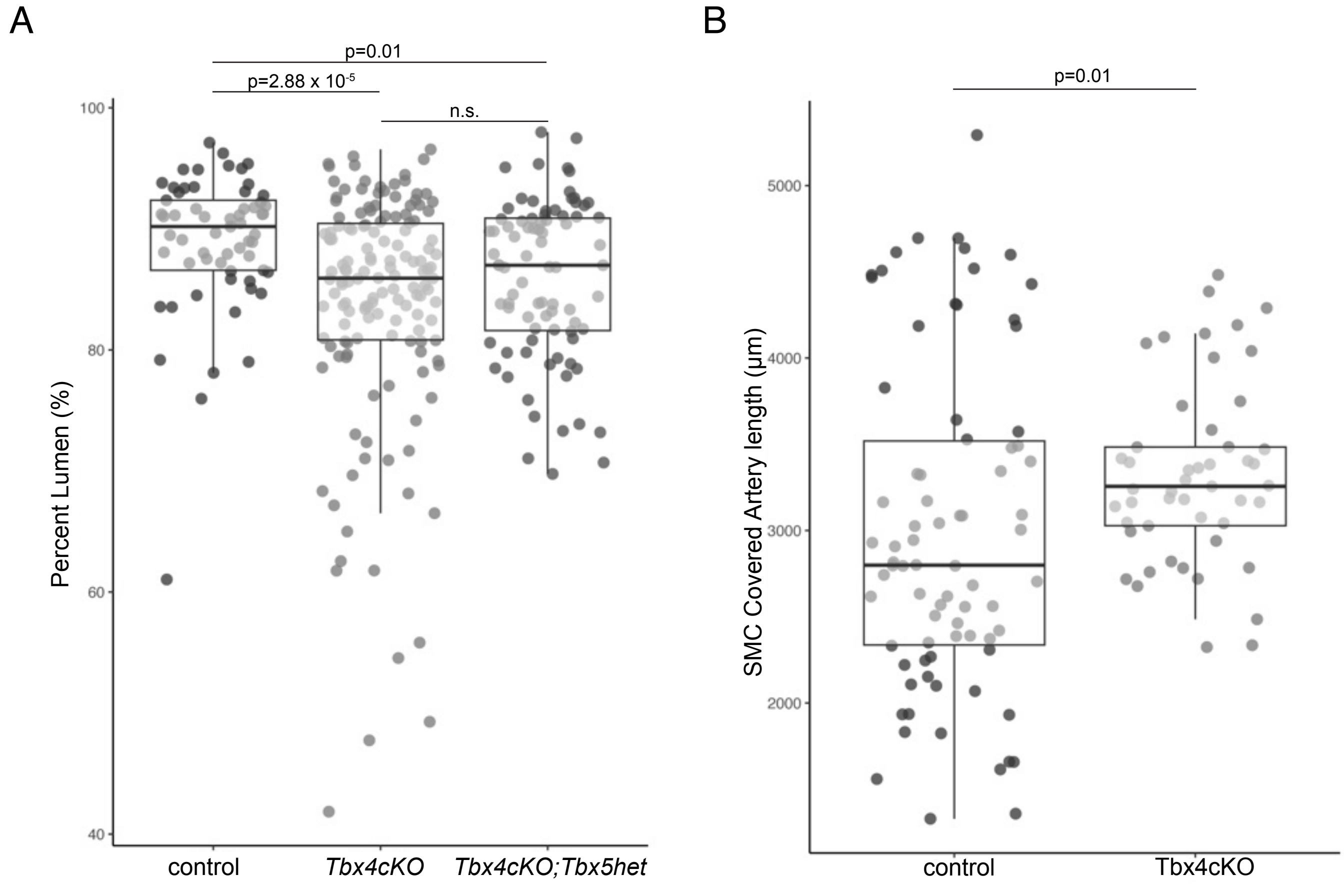

Supplementary Figure 4: Loss of Tbx4 leads to narrowing of artery lumens and extension of artery smooth muscle coat in peripheral vessels. A, diameter of artery lumen is significantly reduced in *Tbx4cKO* and *Tbx4cKO;Tbx4het*. Lumen diameter was determined by subtracting medial + neointimal thicknesses from artery diameter measured in stained cryosections as in Figure 3 and is expressed as a function of artery diameter. **B**, length of muscularized arteries exending toward lobe periphery is significantly expanded in *Tbx4cKO*. The arteries accompanying the airway branches shown in Figure 4, RCd.L3, were measured from the position indicated by asterisks in Figure 4 out to each of the distal-most muscularized tips stemming from that artery.

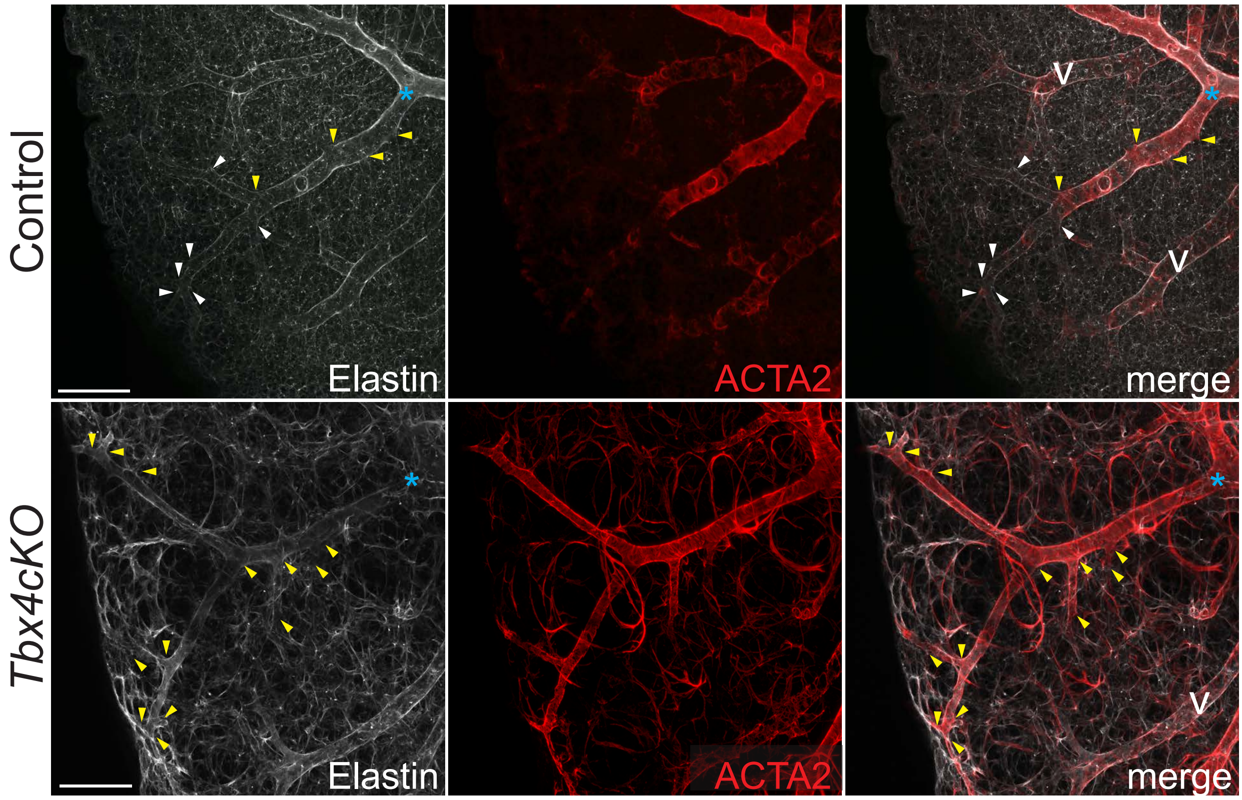

Supplementary Figure 5: *Tbx4cKO* mice exhibit increased arterial branching and distal muscularization. Confocal images of distal arterial branches from whole-mount right caudal lobes stained for elastin (white, hydrazide dye) and smooth muscle actin (red, ACTA2 antibody). Hydrazide dye provides visualization of the elastin-containing vessel outlines regardless of muscularization status, allowing for quantification of both muscularized and non-muscularized branches. *Tbx4cKO* mutants demonstrate increased arterial branching and extended muscularization to the pleural edge compared to controls. Pulmonary arteries and veins were disinguished during quantitation by three diminsional tracing from their respective main vessel. White arrowheads indicate non-muscularized arterial branch points, yellow arrowheads indicate muscularized branch points, blue asterisks mark proximal branch connections to the arterial tree, and V denotes veins. Scale bars: 200 µm.

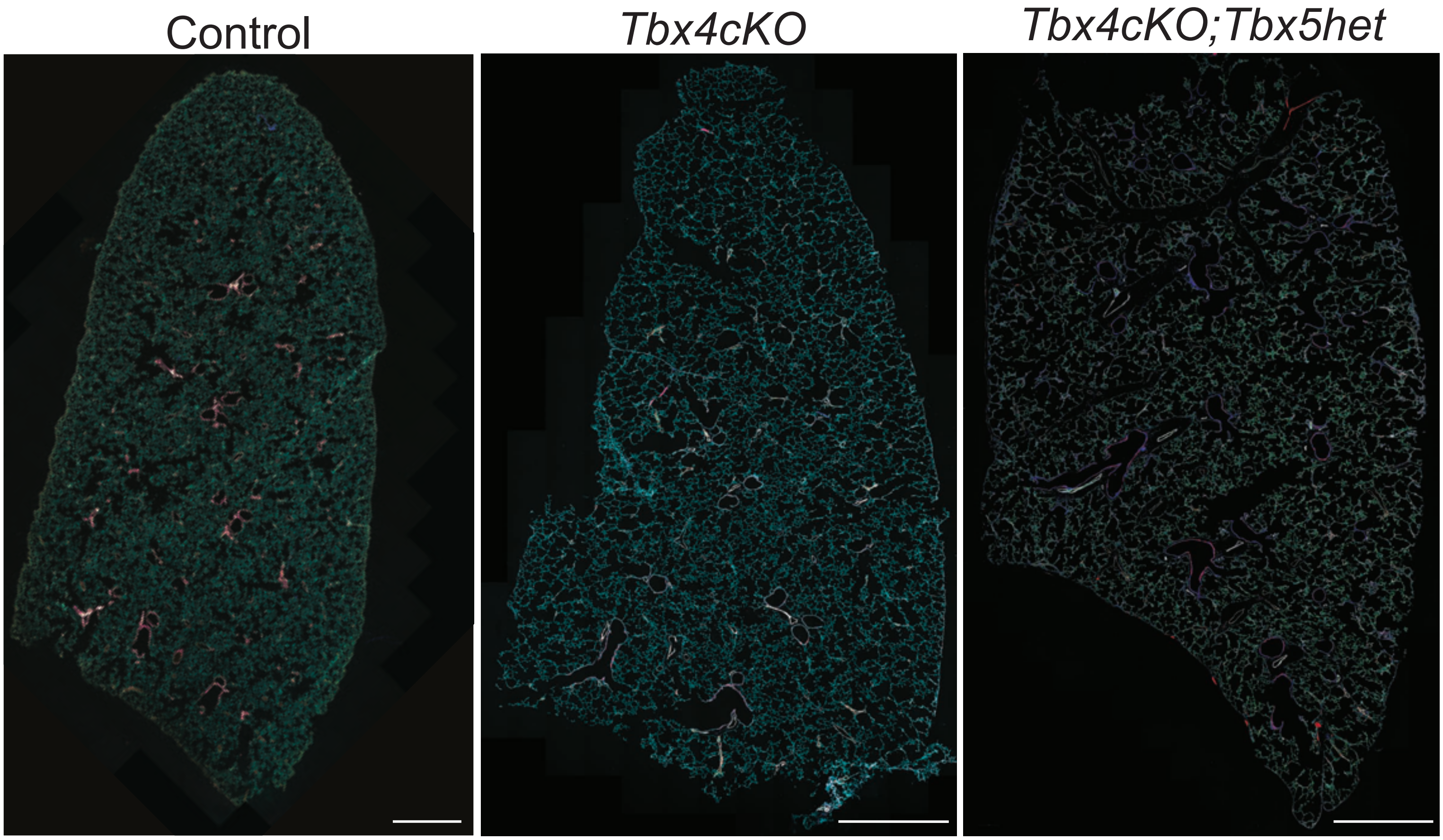

Supplemental Figure 6: Whole section scans of immunostained cryosections for each genotype. Tiled and stitched 25x whole section confocal scans of left lobe cryosections for each genotype. CD31, cyan; ACTA2, red; Hydrazide, white; DAPI (seen in the *Tbx4cKO;TBX5het* scan), blue. Scale bars 500μm

Supplementary Table 1: Comparison of Lung Phenotypes in *Tbx4cKO* Mice and Human TBX4 Syndrome

Feature	Mouse Model (Tbx4cKO)	Human TBX4 Syndrome
**Hemodynamics**		
Pulmonary Hypertension	Present	Present
PH Age of Onset	Adult	Variable (neonatal to geriatric)
**Vasculature**		
Medial Thickening	Present	Present
Neointimal Lesions	Variable	Variable
Distal Muscularization	Present	Present
Muscularized vessels near pleura	Present	Present
PA Branching Pattern	Elaborated	Not reported
**Development**		
Lobe Volume	Reduced at E18.5	Hypoplastic
Distal Lung Development	Underdeveloped distal airspaces reported[38]	Maldeveloped acini or abnormal alveolar growth
**Airways**		
Distal Muscularization	Present	Present
SMC Ring Spacing	Widened	Not specifically characterized
**Abnormal Mesenchymal Features**		
Ectopic Interstitial "Myofibroblasts"	Present	Present
Subpleural SMC Bands	Present	Present
Heterologous Bone Formation	Not seen	Variable

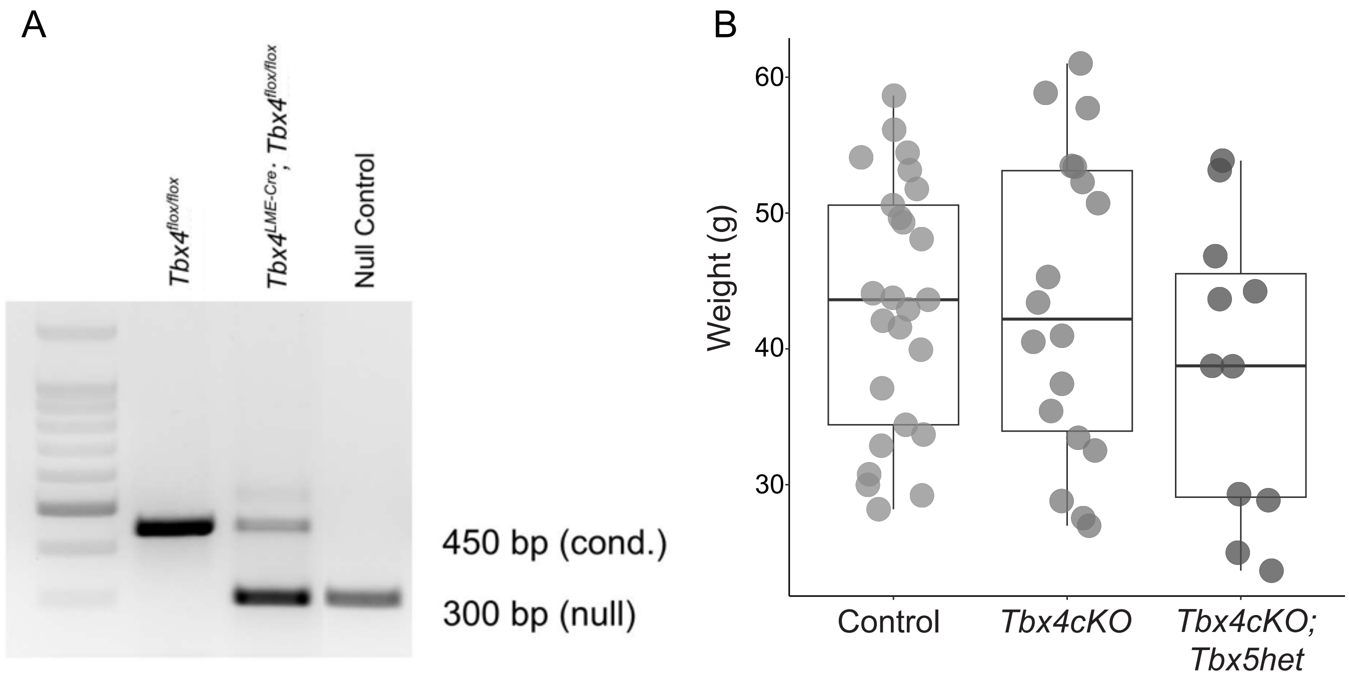

C

PCR primers used to genotype and assess conditional allele excision.

Primer	Sequence
Tbx4 Conditional Allele Excision	
Tbx4-F	GCAGATGACCATCGCTACAA
Tbx4-M	TCATCTAGGCTTCACAGCC
Tbx4-R	GCCAGGCAGAGAAACAAAAG

Supplemental Figure 1. Characterization of Tbx4 deletion in embryonic and postnatal mice. A, Semi-quantitative PCR was performed using three primers targeting the *Tbx4* conditional (“floxed”) and excised conditional (“null”) alleles. The conditional allele was effectively excised in Cre-positive lungs, as indicated by the marked decrease in intensity of the conditional band and presence of the null band. Non-mesenchymal cells carry floxed alleles without Cre expression; therefore, the presence of a conditional band is expected in both *Tbx4cKO* and conditional samples. As a control, the null band was generated using only the two primers targeting the null allele in a *Tbx4cKO* sample. **B**, No significant difference in body weight was found between control, *Tbx4cKO,* and *Tbx4cKO;Tbx5het* adult mice at the time of harvest. **C**, PCR primer sequences (Tbx4-F, Tbx4-M, and Tbx4-R) used for genotyping and verification of conditional *Tbx4* allele excision.

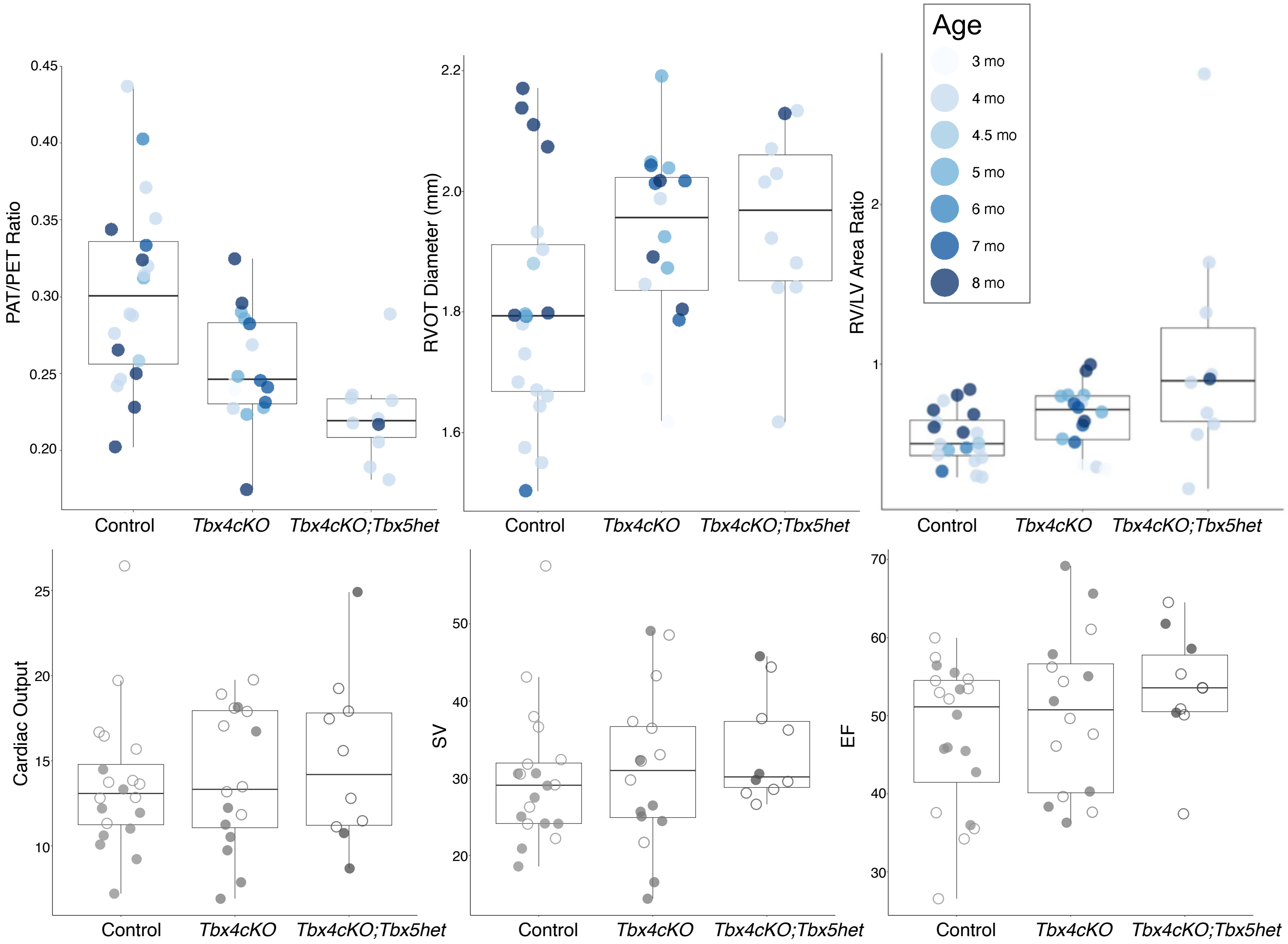

Supplemental Figure 2. Age distribution of right heart measurements and preserved left heart function across genotypes in *Tbx4cKO* and *Tbx4cKO;Tbx5het mice*. Top) PAT/PET ratio, RVOT diameter, and RV/LV area ratio across genotypes, with individual data points color coded by age at measurement. (Bottom) No significant difference in cardiac output (CO), stroke volume (SV), or ejection fraction (EF) between control, *Tbx4cKO*, and *Tbx4cKO;Tbx5het* mice. Statistical comparisons were performed using one-way ANOVA with Tukey's HSD post hoc testing. Males, open circles; females, closed circles.

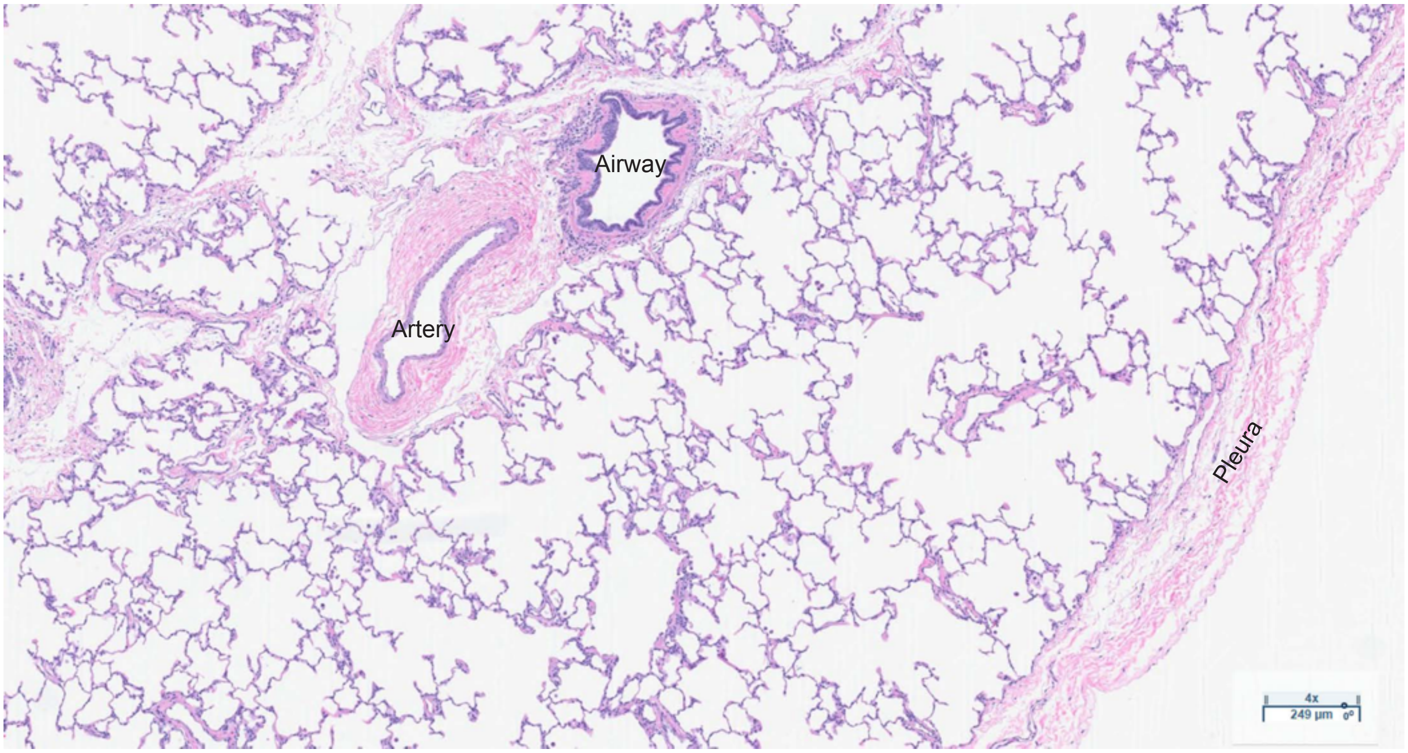

Supplemental Figure 3: Histology of normal human lung. Normal human lung histology demonstrating substantial space between the pleural edge and muscularized airways and arteries. H&E stain of parafin embedded control lung specimen.

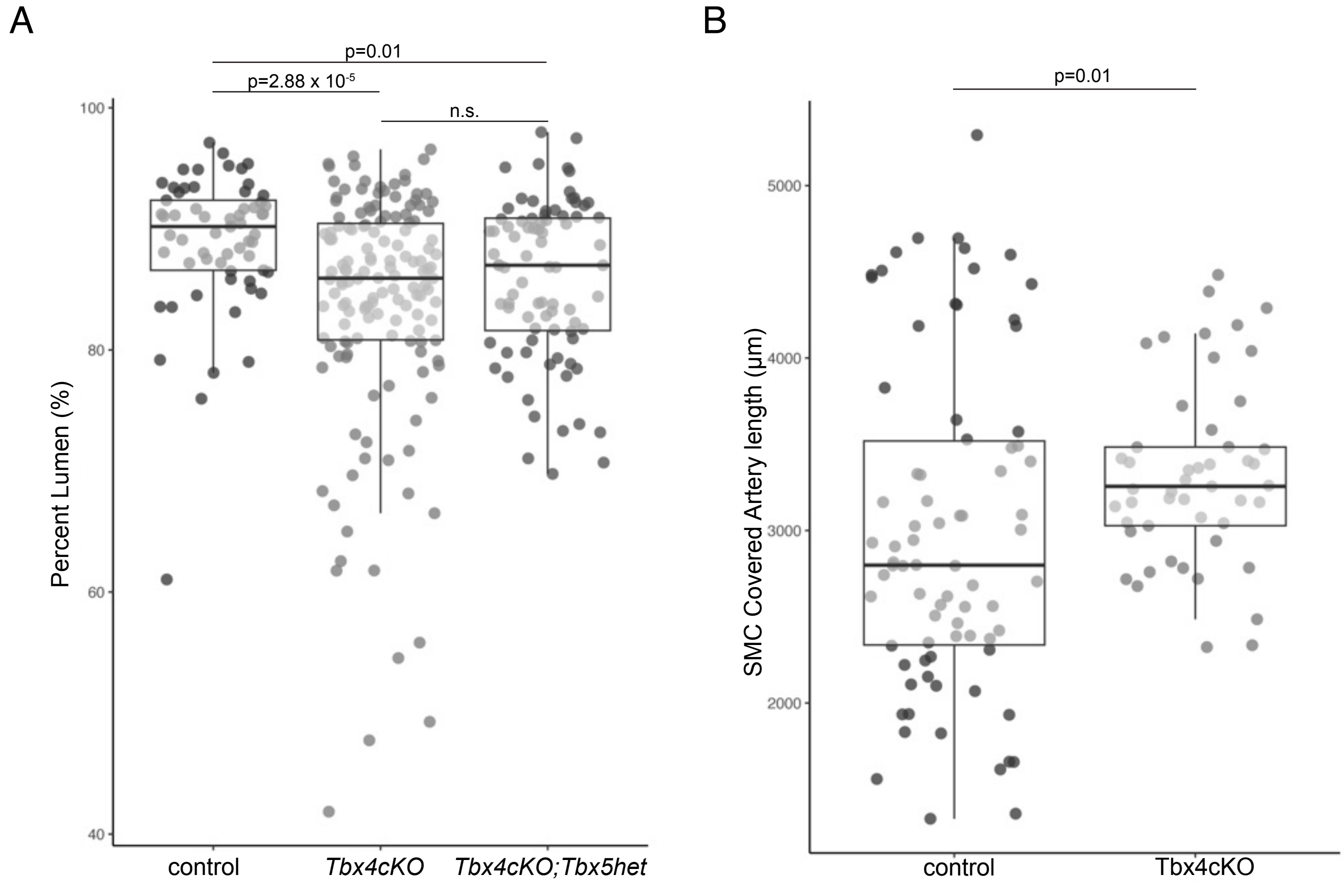

Supplementary Figure 4: Loss of Tbx4 leads to narrowing of artery lumens and extension of artery smooth muscle coat in peripheral vessels. A, diameter of artery lumen is significantly reduced in *Tbx4cKO* and *Tbx4cKO;Tbx4het*. Lumen diameter was determined by subtracting medial + neointimal thicknesses from artery diameter measured in stained cryosections as in Figure 3 and is expressed as a function of artery diameter. **B**, length of muscularized arteries exending toward lobe periphery is significantly expanded in *Tbx4cKO*. The arteries accompanying the airway branches shown in Figure 4, RCd.L3, were measured from the position indicated by asterisks in Figure 4 out to each of the distal-most muscularized tips stemming from that artery.

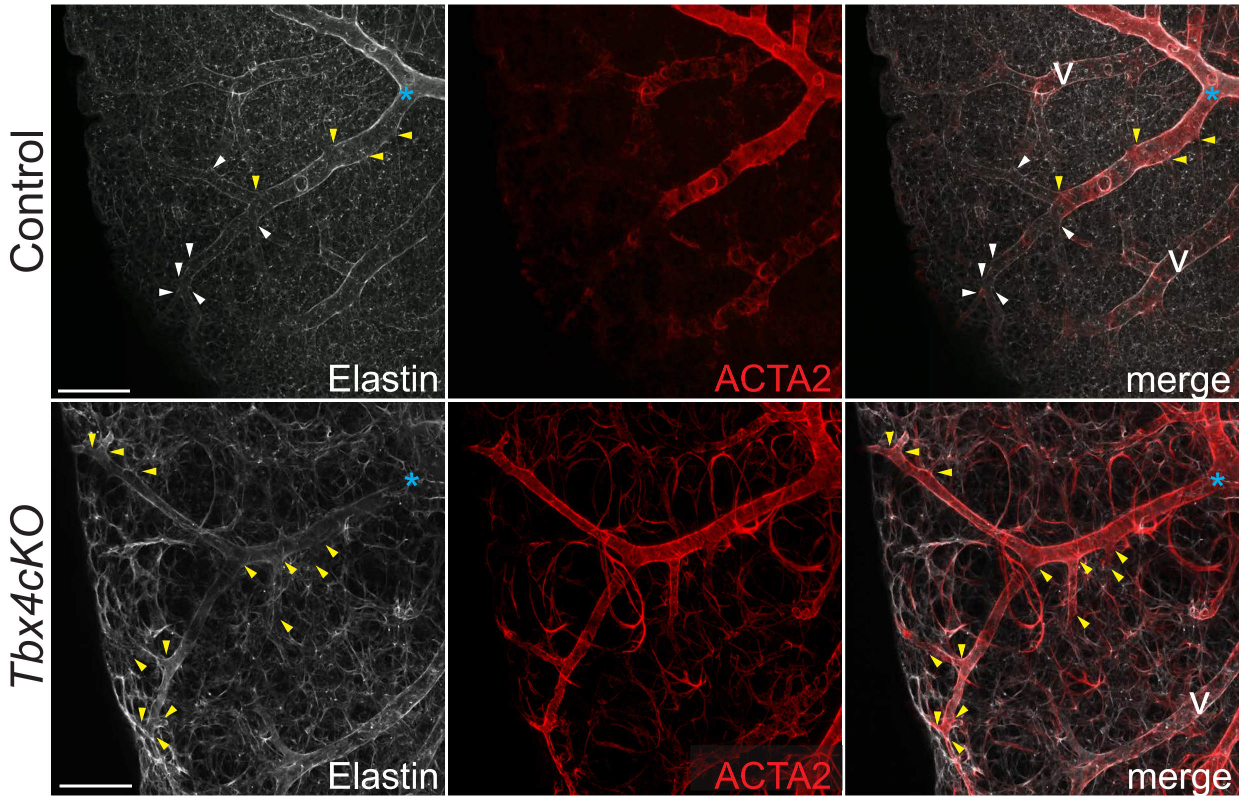

Supplementary Figure 5: *Tbx4cKO* mice exhibit increased arterial branching and distal muscularization. Confocal images of distal arterial branches from whole-mount right caudal lobes stained for elastin (white, hydrazide dye) and smooth muscle actin (red, ACTA2 antibody). Hydrazide dye provides visualization of the elastin-containing vessel outlines regardless of muscularization status, allowing for quantification of both muscularized and non-muscularized branches. *Tbx4cKO* mutants demonstrate increased arterial branching and extended muscularization to the pleural edge compared to controls. Pulmonary arteries and veins were disinguished during quantitation by three diminsional tracing from their respective main vessel. White arrowheads indicate non-muscularized arterial branch points, yellow arrowheads indicate muscularized branch points, blue asterisks mark proximal branch connections to the arterial tree, and V denotes veins. Scale bars: 200 µm.

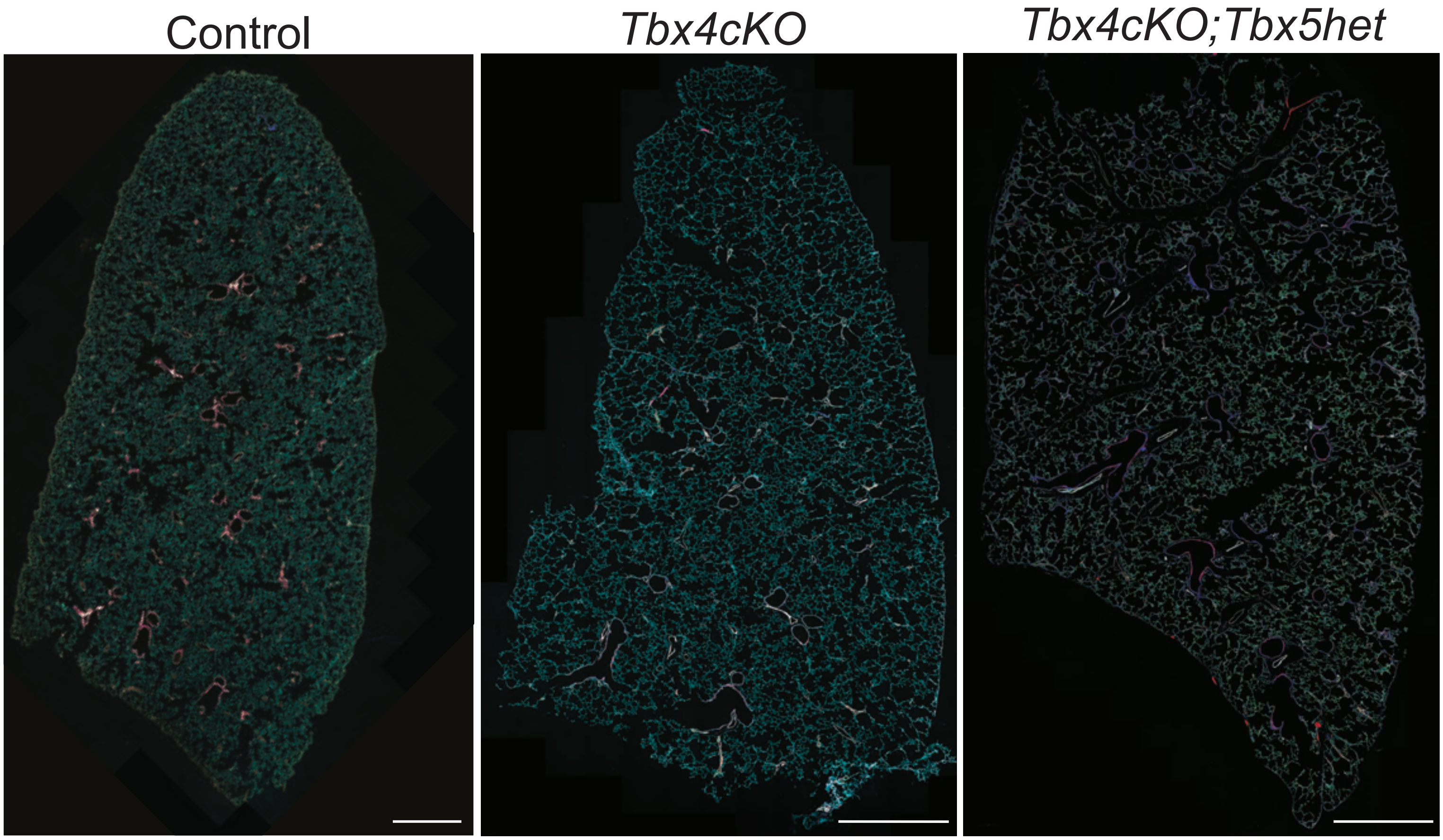

Supplemental Figure 6: Whole section scans of immunostained cryosections for each genotype. Tiled and stitched 25x whole section confocal scans of left lobe cryosections for each genotype. CD31, cyan; ACTA2, red; Hydrazide, white; DAPI (seen in the *Tbx4cKO;TBX5het* scan), blue. Scale bars 500μm
