# Supplementary figures and images for: Excessive postnatal smooth muscle differentiation in a lung-specific model of TBX4-related pulmonary hypertension
Source: JCI Insight. 2026 Apr 23;11(11):e194251. doi: 10.1172/jci.insight.194251 (PMC13313494; doi:10.1172/jci.insight.194251)

# Full Unedited Gel for Supplemental Figure 1

Lanes shown in SupFig.1 indicated with a red asterisk (\*)

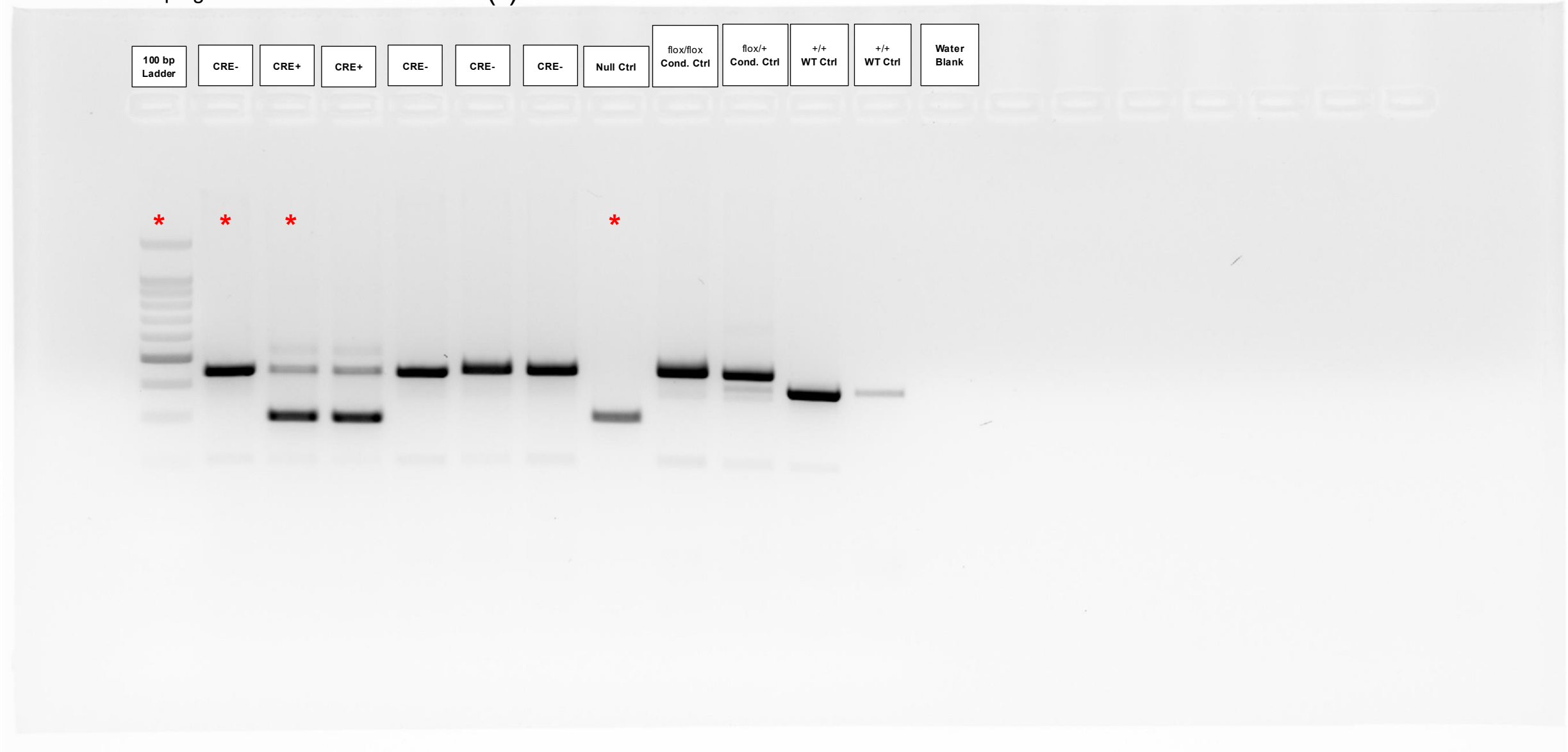

Supplement: Unedited blot and gel images [file jciinsight-11-194251-s208.pdf]
